# Adipokinetic hormones and their G protein-coupled receptors emerged in Lophotrochozoa

**DOI:** 10.1038/srep32789

**Published:** 2016-09-15

**Authors:** Shizhong Li, Frank Hauser, Signe K. Skadborg, Stine V. Nielsen, Nikolaj Kirketerp-Møller, Cornelis J. P. Grimmelikhuijzen

**Affiliations:** 1 Center for Functional and Comparative Insect Genomics, Department of Biology, University of Copenhagen, DK-2100 Copenhagen, Denmark

## Abstract

Most multicellular animals belong to two evolutionary lineages, the Proto– and Deuterostomia, which diverged 640–760 million years (MYR) ago. Neuropeptide signaling is abundant in animals belonging to both lineages, but it is often unclear whether there exist evolutionary relationships between the neuropeptide systems used by proto- or deuterostomes. An exception, however, are members of the gonadotropin-releasing hormone (GnRH) receptor superfamily, which occur in both evolutionary lineages, where GnRHs are the ligands in Deuterostomia and GnRH-like peptides, adipokinetic hormone (AKH), corazonin, and AKH/corazonin-related peptide (ACP) are the ligands in Protostomia. AKH is a well-studied insect neuropeptide that mobilizes lipids and carbohydrates from the insect fat body during flight. In our present paper, we show that AKH is not only widespread in insects, but also in other Ecdysozoa and in Lophotrochozoa. Furthermore, we have cloned and deorphanized two G protein-coupled receptors (GPCRs) from the oyster *Crassostrea gigas* (Mollusca) that are activated by low nanomolar concentrations of oyster AKH (pQVSFSTNWGSamide). Our discovery of functional AKH receptors in molluscs is especially significant, because it traces the emergence of AKH signaling back to about 550 MYR ago and brings us closer to a more complete understanding of the evolutionary origins of the GnRH receptor superfamily.

Adipokinetic hormone (AKH) is an insect neuropeptide produced by the *corpora cardiaca*, two neurohemal organs, often fused, that are closely situated to the insect brain. AKH mobilizes carbohydrates and lipids from the insect fat body (an organ comparable to the mammalian liver and adipose tissue) during high physical activities, such as insect flight and locomotion^1^, but it is also involved in carbohydrate homeostasis in insect larvae^2^,^3^,^4^. Thus, although not structurally related, AKH has the same functions in insects as glucagon and adrenalin have in mammals^5^.

AKH was one of the first neuropeptides to be purified and sequenced from insects and the structure of the first insect AKH from locusts^6^ is shown in Table 1. Since then, AKHs have been isolated from the *corpora cardiaca* of a large number of insect species and turned out to have rather variable structures. From the collection of 32 AKHs listed in Table 1 of Gäde *et al*.^1^ and 13 AKHs listed in Table 1 of Gäde and coworkers^7^ the following hallmarks for AKHs can be deduced: (i) A length of 8, 9, or 10 residues; (ii) a pQ group in position 1; (iii) an aliphatic or aromatic amino acid residue in position 2; (iv) FS, FT, or YS residues in positions 4 and 5; (v) a W residue in position 8; and (vi) either a Wamide, WGamide, or WGXamide C terminus (see also Table 1). We propose to call these AKH peptides “true AKHs”. During genomic and EST database mining, we discovered other AKHs that were 10 amino-acid residues long and that had a WXGamide or WXPamide C terminus (see Table 1 of our current paper). We assign those peptides as “AKH-like”. A third group of AKH-resembling peptides are the “proto-AKHs”. They are longer than 10 amino acid residues, resemble AKHs, but have only 2–4 of the above-mentioned AKH hallmarks (see Table 1).

In 1998, we cloned a G protein-coupled receptor (GPCR) from *Drosophila melanogaster* that was structurally closely related to the mammalian GnRH receptor^8^. Four years later, we isolated the ligand for this receptor from *Drosophila* larvae, which to our surprise was not a GnRH-like peptide, but *Drosophila* AKH^9^. This discovery was the first finding indicating that an evolutionary link exists between insect AKH and vertebrate GnRH signaling. Two other insect receptors are structurally closely related to the insect AKH receptors, which are the corazonin and the AKH/corazonin-related peptide (ACP) receptors^10^,^11^,^12^,^13^. Also the ligands, corazonin and ACP, share several identical and conserved amino acid residues with AKH, suggesting receptor-ligand co-evolution of these three signaling systems^10^,^11^,^12^,^13^. These findings also implicate that all three insect receptors are evolutionarily closely related to the vertebrate GnRH receptors.

The insect AKH receptors are specific for AKH and are not activated by ACP or corazonin. Similarly, the ACP receptors are specific for ACP and the corazonin receptors are specific for corazonin, showing that AKH, ACP, and corazonin are three independent signaling systems^12^,^13^,^14^.

The actions of corazonin in insects are somewhat different from those of AKH. Corazonin was originally isolated from the cockroach *Periplaneta americana*, because of its cardioexcitatory actions on the isolated cockroach heart^15^. However, only in a few insect species such as *P. americana* and *Rhodnius prolixus* this peptide acts excitatory on the isolated insect hearts^15^,^16^. In other insects, such as *D. melanogaster*, corazonin acts as a regulator of insulin-producing cells in the brain^17^, and as a coordinator of sperm transfer and copulation length during mating^18^. In locusts, corazonin induces body melanization, which is characteristic for crowding locusts and associated with pre-swarming behavior^19^. In moths, corazonin is one of a group of hormones that initiates ecdysis^20^. It is difficult to find a common denominator for all these activities, but the actions of corazonin might all be related to starvation and stress^21^,^22^.

So far, the actions of ACP are unknown. It does not mobilize lipids and carbohydrates or has cardioexcitatory effects on the isolated hearts of *R. prolixus*^16^.

**A**KH, ACP, and corazonin and their GPCRs do not always occur together in all insects. For example, mosquitoes (Diptera) have AKH, ACP, and corazonin signaling, while the fruitfly *D. melanogaster* (Diptera) lacks the ACP system and the beetle *Tribolium castaneum* (Coleoptera) lacks corazonin signaling^13^. We hypothesize, therefore, that the three hormonal systems might be back-up systems for each other and that in species, where there is no need for a highly controlled response to starvation or stress, one of the hormonal systems could be superfluous.

Most multicellular animals belong to two evolutionary lineages, the Proto- and Deuterostomia (Fig. 1). In the deuterostome lineage, which comprises all vertebrates and a few invertebrate groups, various forms of GnRH peptides occur together with their GPCRs^23^. In the protostome lineage, which comprises all large groups of invertebrates, AKH, ACP, corazonin and, possibly, some ancestral forms of GnRHs occur together with their GPCRs^23^. The two evolutionary lineages diverged 640–760 MYR ago^27^, meaning that GnRH and its GPCR must have originated before those dates (Fig. 1).

From an evolutionary and comparative endocrinology point of view, it would be highly important to establish when AKH, ACP, and corazonin evolved in the protostome lineage (Fig. 1). Today, this question could perhaps be answered with a certain precision, because the genomes from many protostome invertebrates have been sequenced or are in the process of being sequenced and, also, because high quality Expressed Sequence Tag (EST) databases are available for many protostome invertebrates. Recently, using bioinformatics tools such as TBLASTN screening and phylogenetic tree analyses, we have found that ACPs and their receptors are confined to the Arthropoda^26^.

The situation for corazonin is not as clear as that for ACP. Using TBLASTN searches, corazonin-resembling peptides can be identified in molluscs and annelids, but these molecules also have structural features of GnRH and it is difficult to decide whether they belong to the corazonin or GnRH signaling pathways^26^.

Tracing the evolution of AKH and its receptors appears to be more promising. Using bioinformatics, we and others annotated peptides in molluscs, tardigrades, and penis worms (highlighted with blue in Fig. 1) that were structurally true AKHs^25^,^26^ (Table 1). We also annotated GPCRs in several molluscs and annelids that were promising candidates for being the GPCRs for these AKHs^26^.

In our present paper, we have cloned these predicted molluscan GPCRs from the oyster *Crassostrea gigas* (Bivalvia, Mollusca), expressed them in Chinese Hamster Ovary (CHO) cells and identified (deorphanized) them as being AKH receptors. Our work, therefore, establishes for the first time that functional AKH/AKH-receptor pairs already occur in molluscs. This discovery represents an advancement in invertebrate endocrinology and evolutionary biology, because during nearly forty years^6^ AKH signaling has been regarded as being arthropod-specific.

## Results

### Cloning of the *C. gigas* AKH peptide gene

We have previously annotated the *C. gigas* AKH peptide gene^26^. Using PCR and specific AKH preprohormone primers (Supplementary Table S1) we have now cloned the preprohormone cDNA to establish its correct structure (Supplementary Fig. S1). This preprohormone contains a signal peptide sequence, an AKH sequence, which in its processed (mature) form is pQVSFSTNWGSamide (Table 1), and a C-terminal prohormone sequence, which has a cystine bridge (Supplementary Fig. S1). The mature *C. gigas* AKH sequence is ten amino acid residues long and contains all the hallmarks of insect AKHs. This AKH peptide is very similar to locust AKH-1 (Table 1), because the two peptides share five identical amino acid residues (at positions 1, 4, 7, 8, 9) and three conserved residues (at positions 2, 5, and 10), while the remaining two residues (at positions 2 and 4) are not part of the hallmarks of insect AKH.

### Cloning of the *C. gigas* AKH receptor gene

Using several bioinformatics tools, we recently identified a possible AKH receptor gene in the sequenced genome from *C. gigas*^26^. This gene has previously been cloned by another research group, but was, however, regarded as being an oyster GnRH receptor gene^28^,^29^. In our present paper we have repeated our earlier bioinformatic analyses and could, by lowering the stringencies of our TBLASTN searches, annotate an additional putative AKH receptor gene, showing that two potential AKH receptor genes exist in the oyster. The cDNAs of the first receptor gene, AKHR1, were cloned, which showed that there were four splice variants, A-D, originating from altogether eight exons of the receptor gene (Fig. 2). Three of the four splice variants have been cloned previously (variants A-C of Fig. 2), but were named GnRH receptor splice variants^28^,^29^. Each of our four splice variants coded for a seven transmembrane receptor. An amino acid sequence alignment of the four splice variants is shown in Fig. 3.

The second receptor gene (AKHR2) was cloned as well and this gene contained six exons, yielding a single transcript (Fig. 4). An alignment of its receptor amino acid sequence with the four splice variants of AKHR1 is shown in Fig. 3.

The amino acid sequence encoded by exon 2 of the AKHR2 gene (Fig. 4) strongly resembles the amino acid sequence encoded by exon 3 (Fig. 3) of the AKHR1 gene (68% amino acid residue identity). Similarly, exons 4 and 5 of the AKHR2 gene strongly resemble exons 5 and 6 of the AKHR1 gene, suggesting that the two genes are evolutionary closely related.

We checked whether the two genes were located in the vicinity of each other in the genome of *C. gigas*. Unfortunately this was not possible to determine, as the two genes were located on two scaffolds that were not overlapping.

### Functional expression of the receptor cDNAs

We stably expressed all four cloned splice variants from *C. gigas* AKHR1 (Fig. 2) in Chinese Hamster Ovary (CHO) cells. These cells were also stably transfected with the promiscuous G protein, G-16, and transiently transfected with apoaequorin. An activation of the receptor by its ligand in these pretreated CHO cells would result in an IP_3_/Ca^2+^ second messenger cascade, which could be measured as a bioluminescence signal^30^. We found that splice variants A (AKHR1-A) and B (AKHR1-B) could be activated by low concentrations of *C. gigas* AKH (AKHR1-A, EC_50_ = 4 × 10^−9^ M; AKHR1-B, EC_50_ = 3 × 10^−8^ M), while splice variants C and D, to our surprise, could not be activated (Fig. 5). These results deorphanize the *C. gigas* AKH receptor variants -A and -B, while the variants -C and -D remain orphans.

All four AKHR1 splicing variants could not be activated by *C. gigas* corazonin/GnRH^26^ or other invertebrate neuropeptides (Fig. 5A,C). Insect ACPs, however, are equally potent as *C. gigas* AKH in stimulating the *C. gigas* AKHR1-A and B receptors (Fig. 5A,C, Supplementary Fig. S2). Because ACPs do not occur in *C. gigas*, or in molluscs in general, these findings do not have any physiological implications for the oyster.

The receptor encoded by the second receptor gene (*C. gigas* AKHR2; Fig. 4) could not be activated by *C. gigas* AKH, corazonin/GnRH, or other invertebrate neuropeptides present in our tested peptide library. This receptor, therefore, remains an orphan.

### Genomic organizations of protostome AKH and AKH receptor genes

To further investigate whether the *C. gigas* AKH gene is evolutionarily related to insect AKH genes and whether the *C. gigas* AKH receptor genes are evolutionarily related to the insect receptor genes, we have compared their genomic (intron/exon) organizations. The genomic organizations of the insect AKH preprohormone genes is somewhat variable. These genes possess one or two introns in the coding regions, where one of these introns is often occurring at the same location and having the same intron phasing (indicated by a red zero in Fig. 6). The positions of the other introns are varying (indicated by black numbers in Fig. 6). The genomic organization of the *C. gigas* AKH gene is identical to that of the *Bombyx* mori AKH-1 and -2 genes, sharing one intron with the same intron phasing (Fig. 6). This intron is also present in most of the other insects AKH genes, making the *C. gigas* AKH gene a true member of the family of the established protostome AKH genes. It should be mentioned, however, that also the insect corazonin gene has a similar genomic organization (Fig. 6).

The genomic organization of the coding regions of the insect AKH receptor genes is more complex than that of the AKH peptide genes, comprising 4–6 introns (Fig. 7). The *C. gigas* AKHR gene has two introns in common with the honeybee *Apis mellifera* and mosquito *Anopheles gambiae* AKHR genes and one intron with the *Drosophila melanogaster* and *Bombyx mori* AKHR genes. These introns also have the same intron phasings (Fig. 7). These results strongly suggest an evolutionary relation between the molluscan and insect receptors. Surprisingly, the second *C. gigas* receptor gene, AKHR2 (Fig. 3), which does not code for an AKH receptor, has a genomic organization that is identical to that of the *C. gigas* AKHR1 gene (Fig. 7) although it does not yield splice variants. This suggests a strong evolutionary relationship between AKHR1 and AKHR2. The AKH receptor genes from annelids will be discussed below. Fig. 7 also shows that the human GnRH receptor gene is evolutionarily related to the insect, molluscan, and annelid AKH receptors.

### AKHs and AKH-like peptides present in Ecdysozoa and Lophotrochozoa

We and others have previously shown that AKH peptides similar to *C. gigas* AKH occur in other molluscs and Table 1 gives an updated list that also includes several newly discovered lophotrochozoan and ecdysozoan AKHs. For example, we discovered a novel AKH from the nematode *Globodera rostochiensis* that is C-terminally amidated (Table 1) in contrast to all other AKH-like peptides identified in nematodes, so far, which lack an amidation^31^. We also identified several novel molluscan AKHs (Table 1). The preprohormones of the AKH peptides described in Table 1 are given in Supplementary Fig. S4.

Figure 8 shows an updated phylogenetic tree of the molluscan AKH receptors and other related receptors, which now also includes the four *C. gigas* AKHR1 splice variants (Figs 2 and 3), and the orphan AKHR2 receptor (Fig. 4). Our data suggest that “true” AKHs and AKHRs occur in various molluscs, both bivalves and gastropods (Table 1; Fig. 8).

What about the other Lophotrochozoa? Annelids are an important phylum belonging to the superphylum of Lophotrochozoa. We screened all the genomic and EST libraries available for annelids and could identify a true AKH (pQIHFSPTWGSamide) in the EST database from the thermophile deep-sea annelid *Alvinella pompejana*. However, when we compared the *A. pompejana* AKH preprohormone sequence with other protostome sequences in the NCBI databases using TBLASTN, we found that it was completely identical to the molluscan *Lottia gigantea* AKH preprohormone sequence. This surprising finding must mean that the *A. pompejana* EST database^32^ (http://blast.ncbi.nlm.nih.gov/Blast.cgi) is contaminated with *L. gigantea* cDNA.

Veenstra^33^ annotated several peptides from the annelids *Capitella teleta* and *Helobdella robusta*, which he named GnRHs (Table 1). Some of these peptides, however, have several of the hallmarks of AKHs and would be better assigned to as being proto-AKH peptides (Table 1). We annotated an additional proto-AKH from *C. teleta* and two proto-AKHs from the annelid *Platynereis dumerilii* (Table 1). All these annelid proto-AKH peptides are 12–14 amino acid residues long and have structural properties of AKH. For example, *C. teleta* GnRH-2 and *P. dumerilii* proto-AKH-1, which have the same sequence pQFSFSLPGKWGNamide (Table 1), have: (i) a blocked (pQ) N terminus (ii) an aromatic residue (F) in position 2; (iii) an FS sequence in position 4 and 5; and (iv) a WGNamide C terminus, which are all hallmarks for AKHs. The only differences with genuine AKHs are their sizes (12 instead of 8 or 10 residues), and a lack of a W residue at position 8, although a W residue is present at position 10.

The other annelid peptides listed in Table 1 resemble AKHs, but all to a lesser extent than *C. teleta* GnRH-2 and *P. dumerilii* proto-AKH-1. We were unable to identify true AKH peptides in the genomic and EST databases (http://blast.ncbi.nlm.nih.gov/Blast.cgi) from annelids.

Also in brachiopods we find an AKH-resembling peptide that is longer than 10 amino residues, has three of the AKH hallmarks and that we, therefore, assign as a proto-AKH (Table 1).

**Figure 1:**
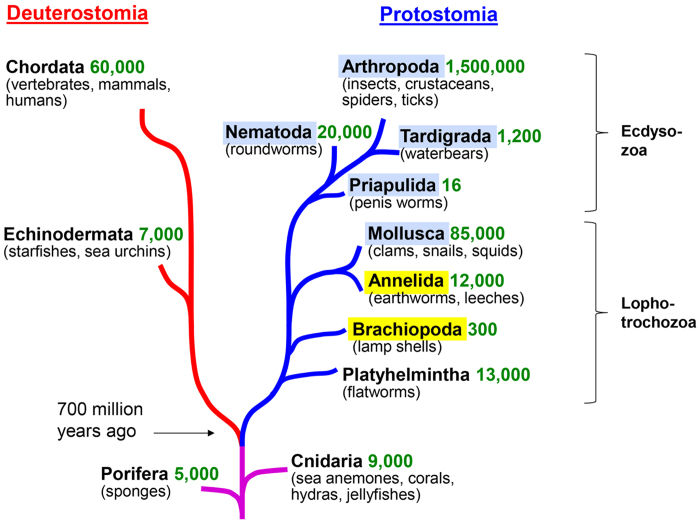
A simplified phylogenetic tree of multicellular animals, showing two evolutionary lineages of animal evolution: the Protostomia (blue line) and the Deuterostomia (red line). Furthermore, two phyla, Cnidaria and Porifera, are shown that emerged before the split of Proto- and Deuterostomia (violet line). Species numbers are given in green. Phyla in which AKHs occurs are highlighted in light-blue. Phyla in which proto-AKHs occur are highlighted in yellow.

**Figure 2:**
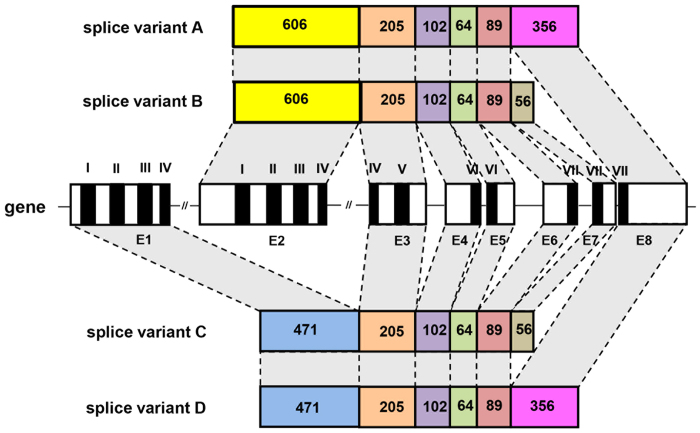
A schematic representation of the *C. gigas* AKH receptor-1 (AKHR1) gene and the four splice variants that we identified. The gene has eight exons (E1 to E8) that yield splice variants A-D. The numbers given in each exon are the numbers of nucleotides. The roman numbers indicate the positions of the DNA sequence that code for the transmembrane regions (I-VII) of the receptor protein.

The phylum Platyhelmintha is another important group within the Lophotrochozoa (Fig. 1). We were, however, unable to find AKH or AKH-like sequences in any of the following platyhelminths with available whole genome sequence data: *Clonorchis sinensis*, *Echinococcus multioccularis*, *Fasciola hepatica*, *Gryodactus salaris*, *Opistorchis viverrini opera*, *Schistostoma haematobium*, *Schistostoma mansoni*, and *Schmidtea mediterrana,* using TBLASTN screening. Also the EST databases from these platyhelminths did not give any positive hits. It appears, therefore, that platyhelminths do not have AKHs. For the other lophotrochozoans are, so far, no genomic databases available.

### AKH receptors in Ecdysozoa and Lophotrochozoa

We screened the genomic databases of selected ecdysozoans and all lophotrochozoans that have a sequenced genome for the presence of AKH and ACP receptors. Figure 8 shows two clusters of AKH receptors, one comprising the arthropod receptors and the other comprising the annelid and molluscan receptors. The established *C. gigas* AKHR1-A and AKHR1-B receptors (highlighted in bold) are lying very close to each other, which is in accordance with both being genuine AKH receptors (Fig. 5).

The ACP receptors form a separate cluster that can only be found in Arthropoda (Fig. 8), confirming that ACP signaling only occurs in Arthropoda and not in other ecdysozoans and lophotrochozoans.

## Discussion

AKH was one of the first insect neuropeptides to be sequenced^6^ (Table 1). During the nearly forty years that followed, a very large number of AKHs was isolated from insects and other arthropods^1^, which established the view, among invertebrate endocrinologists, that AKH was an arthropod-specific neurohormone. Only in the last few years, after the completion of several non-arthropod genome projects, it was realized that AKHs could also occur in other, non-arthropod invertebrates^23^,^24^,^25^,^26^,^27^,^34^. In our current paper we have deorphanized two AKH receptors, AKHR1-A and -B (Fig. 5), from the oyster *C. gigas* (Mollusca). These results show for the first time that AKH signaling indeed occurs in molluscs, which represents a milestone in AKH research, because all the knowledge of arthropod AKH biology might now be extended to members of the Lophotrochozoa and contribute to our understanding of the physiology of this important group of invertebrates.

So what could the function of AKH be in, for example, oysters? From insects it is known that AKH is involved in carbohydrate homeostasis and that it is mobilizing sugars from the fat body and acting as a counterpart of insulin^1^,^2^,^3^,^4^. It would be worthwhile to investigate this action of AKH in molluscs, especially because several insulins are known to be present in members of this phylum^35^,^36^,^37^,^38^.

Furthermore AKH in insects mobilizes carbohydrates and lipids under conditions of physical stress such as flight and longterm locomotion^1^,^2^,^3^,^5^. Oysters and other bivalves are often exposed to periods of anoxia, because of tide changes in their habitats. Under these anaerobic conditions AKHs could perhaps initiate glycogen breakdown, thus enabling ATP production in the animal by anaerobic glycolysis. This would certainly be a very useful strategy of the animal to survive longterm anoxia.

Johnson *et al*.^34^ have recently shown that an AKH is present in the sea slug *Aplysia californica*. Injection of this peptide into the slug appears to reduce feeding and induce defecation, while it has no influence on hemolymph glucose concentrations^34^. These findings are difficult to reconcile with our above considerations, but our own considerations are, of course, only hypothetical.

**Figure 3:**
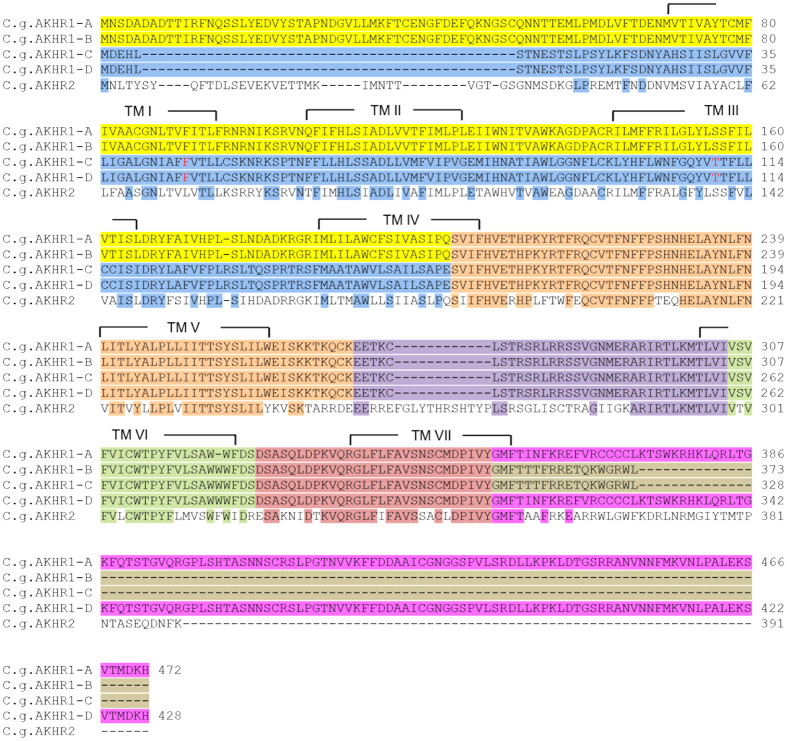
Alignment of the amino acid sequences of the four splice variants of *C. gigas* AKH receptor-1 (AKHR1). The same color code is used as in Fig. 2, meaning that the amino acid residues highlighted with one color (Fig. 3) are coded for by the exon highlighted in the same color (Fig. 2). The transmembrane regions are indicated by TM I-TM VII. Also the AKHR2 is aligned with AKHR1-D. Identical amino acid residues between AKHR2 and AKHR1-D are highlighted in the colors corresponding to each exon of the AKHR1 gene. The highlighted F residue at position 46 of *C. gigas* AKH1-C was an S residue in the reference *C. gigas* genome sequence. Similarly, the highlighted T residue at position 110 of AKH1-C was an A residue in the reference *C. gigas* genome sequence. These changes are probably allelic variations. The cloned cDNA sequences were submitted to GenBank with the following accession numbers: *C. gigas* AKHR1-A, KM205066; *C. gigas* AKHR1-B, KM205067; *C. gigas* AKHR1-C, KM205068; *C. gigas* AKHR1-D, KM205069; *C. gigas* AKHR2, KM205070.

**Figure 4:**
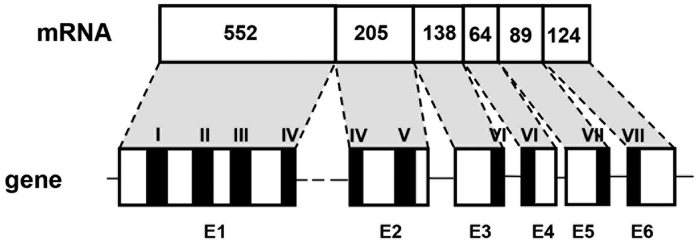
Genomic organization of *C. gigas* AKH receptor-2 (AKHR2) and the mRNA that it codes for. The abbreviations are the same as in Fig. 2.

**Figure 5:**
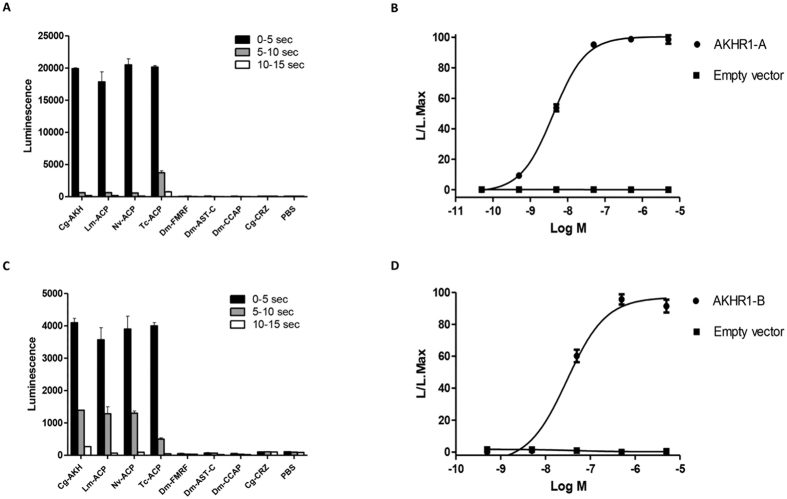
Functional expression of the *C. gigas* AKHR1 splice variants A (AKHR1-A) and B (AKHR1-B) in CHO cells. The vertical bars represent S.E.M. (n = 3), which are sometimes smaller than the symbols or lines used. In these cases only the symbols or lines are given. (**A**) Bioluminescence responses of CHO/G-16/AKHR1-A cells after addition of 10^−6^ M *C. gigas* AKH (Cg-AKH); *Locusta migratoria* ACP (Lm-ACP); *Nasonia vitripennis* ACP (Nv-ACP); *Tribolium castaneum* ACP (Tc-ACP), *D. melanogaster* FMRFamide (Dm-FMRF); *D. melanogaster* allatostatin C (Dm-Ast-C); *D. melanogaster* crustacean cardioactive peptide (Dm-CCAP); *C. gigas* corazonin (Cg-CRZ); and phosphate buffered saline (PBS). The AKHR1-A receptor can be activated by authentic C. gigas AKH, but also by insect ACPs that do not occur in *C. gigas* and other molluscs. Other peptides are not active. (**B**) dose response curve of the effect of *C. gigas* AKH seen in A. The EC_50_ of this activation is 4 × 10^−9^ M. (**C**) A similar experiment as in A, but now using CHO cells stably transfected with the AKHR1-B receptor. (**D**) A similar dose response curve as in B, but now using cells stably transfected with the AKHR1-B receptor.

Identifying AKH and AKH receptors by bioinformatics is not always sufficient to show that AKH signaling occurs in a certain animal group. This is illustrated in this paper by the existence of AKHR1-C and -D and AKHR2 receptors (Fig. 3) that, although structurally similar to AKHR1-A and AKHR1-B (Fig. 8), can not be activated by AKH. Only the demonstration of functional AKH/AKHR pairs such as *C. gigas* AKH and AKHR1-A and -B (Fig. 5) proves that AKH signaling really occurs in oysters.

From our previous experiments in insects we have learned that the AKH receptors are specific for AKH and do not crossreact with corazonin or ACP. Similarly ACP receptors are specific for ACP and do not cross react with AKH or corazonin^12^,^13^. We were, therefore, surprised to see that *C. gigas* AKHR1-A and -B could be activated with equal potencies by *C. gigas* AKH and various insect ACPs, although it was insensitive for *C. gigas* corazonin/GnRH (Fig. 5, Supplementary Fig. S2). There are two explanations for this phenomenon. First, when AKH, ACP, and corazonin signaling co-exist, such as in many insects, there appears to be an evolutionary pressure on each receptor that it only can be activated by its own ligand. When, like in oysters, ACP does not occur, the AKH receptor does not have that evolutionary pressure anymore so that heterologous ACP, which has many similarities to *C. gigas* AKH (Supplementary Fig. S3), is able to activate the *C. gigas* AKH receptor.

The other explanation is that the ACP receptor gene originated from the AKH receptor gene by gene duplication and, similarly, the ACP peptide gene originated from the AKH peptide gene^13^,^26^. These gene duplications probably occurred in early arthropods, because ACP signaling, as explained above, only exists in arthropods. In animals that arose before the emergence of arthropods, such as mollsucs, the binding pockets of the AKH receptors could have structural properties of both the binding pockets of the AKH receptors and ACP receptors, thus allowing binding, in our case, of both AKH and ACP (Fig. 5, Supplementary Fig. S2). The binding pockets of the AKH receptors differentiated into AKH-specific and ACP-specific binding pockets only after the gene duplications lead to separate AKH and ACP signaling. Our findings that ACPs can stimulate the *C. gigas* AKH receptors, therefore, support our theory that ACP and AKH signaling are evolutionarily closely related^13^.

One of the aims of the current study was to determine when AKH signaling emerged during evolution. The answer is that this likely occurred in the Lophotrochozoa. But in which phylum did this happen? We found “true” AKH and “AKH-like” peptides (for a definition see second paragraph of the Introduction, or the legend of Table 1) in all molluscs with a sequenced genome, such as *C. gigas* (Bivalvia), and *Biomphalaria glabrata*, *L. gigantea* (Gastropoda), *A. californica* (Gastropoda), and in several molluscs with EST databases, such as *Bithynia siamensis goniomphalos* (Gastropoda), *Tritonia diomedea* (Gastropoda), *Hyriopsis cumingii* (Bivalvia), *Mitylus galloprovincialis* (Bivalvia), and *Biomphalaria glabrata* (Gastropoda) (Table 1). We also checked the EST libraries of several cephalopods (*Loligo pealeii*, *Nautilus pompilius*, *Sepia officialis*, and *Octopus vulgaris*) (http://neurobase.rc.ufl.edu/cephalopods) but, so far, were unable to identify AKHs, which might suggest that, perhaps, not all molluscs have AKH signaling.

The annelid *C. teleta* (Table 1) produces several peptides that resemble AKHs but that are not AKHs *sensu stricto*. Their sizes are 12 or 14 amino acid residues and they often lack a tryptophan residue at position 8. When we collectively take the arthropod AKHs as a standard for AKH, these annelid peptides are not AKHs. But what are these peptides then? When we look at the structure of these peptides, we can see that they have retained several properties of AKHs. For example, *C. teleta* GnRH-2 and *P. dumerilii* proto-AKH-1 have a pQ group at position 1, an aromatic residue (F) at position 2, an FS sequence in positions 4 and 5, and a WGNamide C terminus (Table 1). These peptides resemble AKH, but they are longer than the standard AKHs. *Capitella teleta* GnRH-2, -2a, and -3 have originally been named GnRHs^33^, but these peptides have only very few amino residues in common with chicken GnRH-1 and -2, or sea urchin GnRH^39^,^40^,^41^ (Supplementary Figs S6 and S7). We, therefore, propose to call the annelid and brachiopod peptides “proto-AKHs”, meaning that they have several structural properties of AKHs (2–4 of the AKH hallmarks mentioned in Table 1) and that they are possibly representing ancestor forms of AKHs. All these annelid peptides are excellent candidates for the annelid AKH receptors shown in Fig. 8.

**Figure 6:**
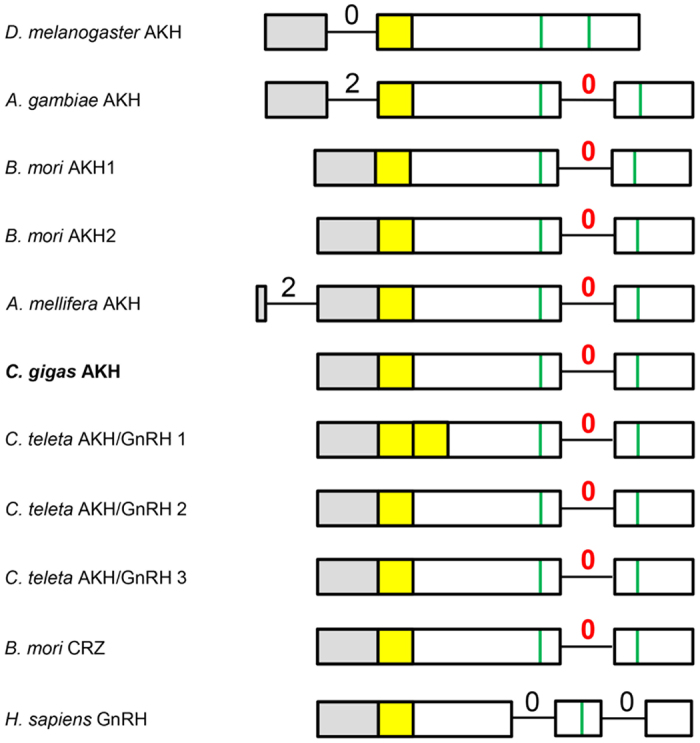
Genomic organizations of the coding regions of the AKH genes from several insects (*D. melanogaster*, *Anopheles gambiae*, *Bombyx mori*, *Apis mellifera*), the mollusc *C. gigas*, and the annelid *Capitella teleta*. For comparison the corazonin (CRZ) gene from *B. mori* and the GnRH gene from *Homo sapiens* are also included. The numbers give intron phasings; red numbers indicate introns in common with the *C. gigas* AKH gene; black numbers indicate those introns that are not in common. Grey highlights the regions in the genes that code for the signal peptide; yellow highlights the regions coding for AKH, GnRH, or CRZ; vertical green lines give the positions coding for those cysteines that form cystine bridges in the AKH and CRZ preprohormones.

**Figure 7:**
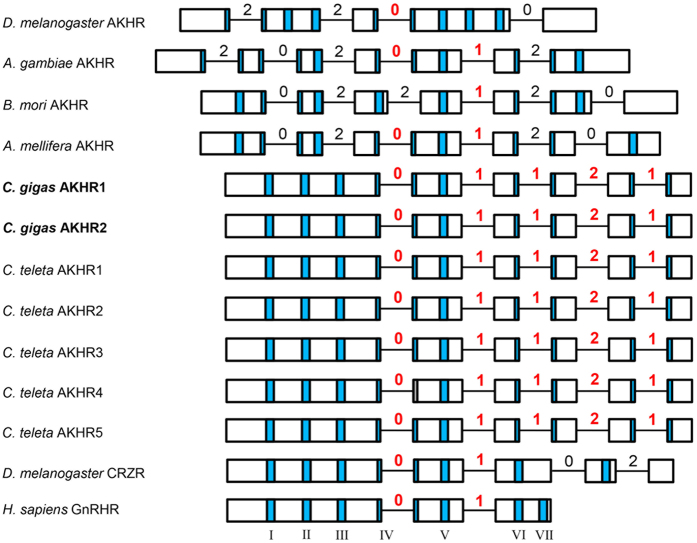
Genomic organizations of the coding regions of the AKH receptor genes from several insects, the molluscs *C. gigas* and the annelid *C. teleta*. For comparison the corazonin receptor (CRZR) gene from *D.* melanogaster and the GnRH receptor (GnRHR) gene from *H.* sapiens are also included. For other abbreviations see Fig. 6. The numbers give intron phasings; red numbers indicate introns that are in common with the *C. gigas* AKHR1 gene; black numbers are introns that are not shared with this gene. Roman numbers (I-VII) indicate the regions that code for the transmembrane regions (given as vertical blue bars).

**Figure 8:**
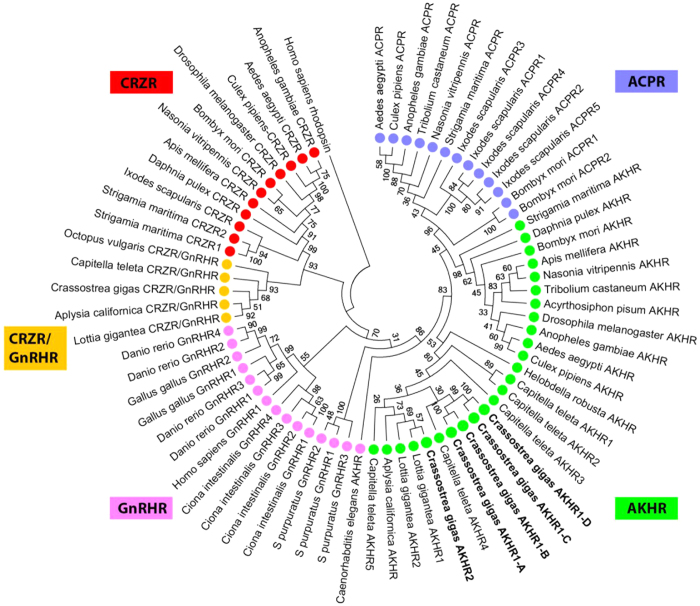
Phylogenetic tree analysis of protostome ACP receptors (indicated with blue dots), protostome AKH receptors (green dots), deuterostome GnRH receptors (pink dots), protostome corazonin/GnRH receptors (yellow dots), and protostome corazonin receptors (red dots). The receptor sequences were retrieved form NCBI and most of them are published (see, for example refs 25,28,29,31,46, 47, 48, 49, 50). See also Supplementary Fig. S5 online for amino acid sequences and accession numbers of the receptors. The receptors cloned in the current paper are highlighted in bold. The MEGA.6.06 software program based on the neighbour-joining calculation method^44^,^45^ was used. The tree is rooted with human rhodopsin. Bootstrap values (1–100) are given at each branch. AKHR = AKH receptor; ACPR = ACP receptor; CRZR = corazonin receptor; GnRHR = GnRH receptor.

Could the genomic organization of the annelid proto-AKHs (Table 1) and the annelid AKH receptors (Fig. 8) perhaps help us in deciding whether they are genuine AKHs or AKH receptors? Figs 6 and 7 show that this is not possible, because the genomic organizations of the annelid proto-AKH peptide genes are identical to both those of the corazonin genes and the AKH genes (Fig. 6). The same is true for the annelid AKH receptor genes, which have an intron/exon organization identical to that of both the corazonin and molluscan AKH receptor genes (Fig. 7).

As mentioned in the Results part, we were unable to find AKH or AKH receptors in platyhelminths (Fig. 1) and the same is true for rotifers and other small phyla belonging to the Lophotrochozoa. Our conclusion, therefore, is that AKH signaling originated in Mollusca or a close ancestor of this phylum. Thus, our work traces the emergence of AKH signaling back to the early Cambrian period, about 550 MYR ago^42^, which is shortly before the emergence of the Arthropoda^43^, where we know that ACP signaling evolved.

## Methods

### Animals and total RNA purification

Adult pacific oysters, *C. gigas*, were cultured at the Atlantic coast of France, and purchased from a local supermarket in Copenhagen (Irma, Denmark). After removal of the shells, the animals were quickly frozen in liquid nitrogen. The whole frozen bodies from five animals were crashed and grinded in a mortar, and 1g of the frozen powder was used for total RNA isolation using an RNeasy^®^ Mini Kit (Qiagen). The remaining genomic DNA was removed using the RNase-Free DNaseI (Qiagen) according to manufacturer’s instructions.

### Reverse transcription polymerase chain reaction (RT-PCR) and PCR

Primers for the PCR amplifications were designed based on the annotated *C. gigas* AKH peptide and AKH receptors (see Supplementary Table S1). All the sense primers for the receptor cloning contain a Kozak consensus sequence, GCCACC, and the sense primers for cloning *C. gigas* AKHR1-A and *C. gigas* AKHR1-B contain a *Pst*I restriction site, while all the antisense primers for the receptor cloning contain a *Sac*II restriction site. Reverse transcriptions were performed on 1 μg total RNA using the SuperScript^®^ III First-Strand Synthesis SuperMix (Invitrogen) according to the manufacturer’s instructions to get the single stranded cDNA, and a control was included without the reverse transcription enzyme. PCR amplifications were carried out using the PCR HotStar Taq Master Mix Kit (Qiagen). The following components were mixed: 1 μl cDNA, 10 μl 2 ×  Hot Star Taq Plus Master Mix, 2 μl sense primer (10 pmol/μl, Eurofins Genomics) and 2 μl antisense primer (10 pmol/μl, Eurofins Genomics) with a total volume of 20 μl in a reaction by adding 5 μl RNase-free H_2_O (Qiagen). The following program was used for the PCR experiment: initial denaturation at 95 °C for 5 min; 94 °C for 30 s; 55 °C for 30 s; 72 °C for 2 min; elongation at 72 °C for 10 min. Samples with no reverse transcriptions were used for the genomic DNA controls. Specificity of the PCR products was analyzed on 1% agarose gel.

### TOPO Cloning for sequence analysis and pIRES2-ZsGreen1 (pIRES) vector subcloning

The amplified products were purified using the Agarose Gel Extract Mini Kit (5 Prime) according to manufacturer’s instructions on 1% agarose gel. The concentration of the DNA purified was measured with Nanodrop (Thermo Scientific). 10–20 ng DNA was mixed with TOPO^®^ vector using TOPO TA cloning^®^ Kit (Invitrogen), and subsequently the ligated vectors were transformed into One Shot^®^ Chemically Competent *E. coli* (Invitrogen) following the manufacturer’s instructions. The plasmid DNA containing the inserts from the selected *E.coli* was purified using Fast Plasmid Mini Kit (5 Prime). The DNA was sequenced by Eurofins Genomics, Germany.

The TOPO plasmids with *C. gigas* AKHR1-A or *C. gigas* AKHR1-B inserts and the empty pIRES- ZsGreen1 vector (Clontech) were subjected to *Pst*I (BioLabs) and *Sac* II (BioLabs) restriction enzyme digestion. The TOPO plasmids with *C. gigas* AKHR1-C, *C. gigas* AKHR1-D or *C. gigas* AKHR2 inserts and the empty pIRES-ZsGreen1 vector were subjected to *EcoR*I (BioLabs) restriction enzyme digestion. The purified inserts were ligated to the digested pIRES vector using the Rapid DNA Dephos and Ligation Kit (Roche), and the ligated vectors were transformed into *E. coli*. Plasmid DNA was prepared from a large-scale culture using the Nucleo Bond^®^ Xtra Midi EF kit (Macherey Nagel) following the manufacturer’s instructions and sequenced.

### Chinese Hamster Ovary (CHO) cell culture, transfection and bioluminescence assay

CHO cells stably expressing the human G-protein 16 (CHO/G16) were maintained in DMEM/F-12 + GlutaMAX^TM^ (Life Technologies) medium with 200 μg/ml hygromycin B (Invitrogen), 5% fetal calf serum (FCS) (Life Technologies), 1% (v/v) glutaMAX (Life Technologies), 100 U/ml penicillin(Life Technologies), and 100 μg/ml streptomycin (Life Technologies). To express the AKH receptor, 5 × 10^4^ CHO/G16 cells were transfected with 3 μg pIRES vector containing the AKH receptor insert using JetPEI^®^ transfection reagent (Polyplus), while 3 μg empty pIRES vector was used as a control. All tested peptides were from Genemed Synthesis, Inc. and initially dissolved in demethylformamide (DMF) followed by dilution in PBS. The purities of the peptides and peptide contents were determined by HPCL and mass spectrometry as supplied by Genemed Synthesis. The bioluminescence assay was performed as described earlier^9^,^30^ using a Victor2 1420 Multilabel Counter (Perkin Elmer).

A selected single cell line from the CHO/G16 cells giving a strong response was further tested for a dose response curve with a serial dilution of the peptides, and the EC_50_ values were subsequently determined.

### Data processing and bioinformatics tools

DNA sequence comparisons were done using CLC Main Workbench Version 6.2 (CLCBio). Protein sequence alignments were carried out using ClustalW2 (http://www.ebi.ac.uk/Tools/msa/clustalw2/). The bioassay data were analyzed and the EC_50_ values were calculated using GraphPad Prism software (Version 5.0).

TBLASTN homology searches were performed in the nucleotide collection (nr/nt), expressed sequence tags (EST), transcriptome shortgun assembly (TSA), and whole-genome shotgun contigs (WGS) databases at NCBI (http://www.ncbi.nlm.nih.gov/) using known AKH and AKHR sequences. Phylogenetic tree analyses were performed using the MEGA.6.06 and Neighbor-Joining methods^44^,^45^. Prediction of transmembrane helices of the receptor proteins was done using the TMHMM server (http://www.cbs.dtu.dk/services/TMHMM/), signal peptides were predicted using the SignalP 4.1 server (http://www.cbs.dtu.dk/services/SignalP/).

## Additional Information

**Accession codes**: The cloned cDNA sequences reported in this paper have GenBank accession numbers KM205066-KM205070, and KM205073.

**How to cite this article**: Li, S. *et al*. Adipokinetic hormones and their G protein-coupled receptors emerged in Lophotrochozoa. *Sci. Rep.* **6**, 32789; doi: 10.1038/srep32789 (2016).

## Supplementary Material

Supplementary Information

## Figures and Tables

**Table 1:**
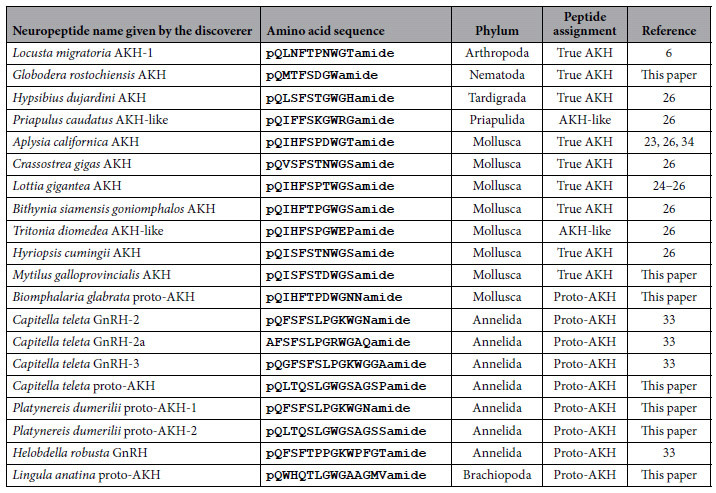
Some of the novel AKH and AKH-like peptides recently identified by us and other research groups.

*Locusta migratoria* AKH-1, the first insect AKH that was isolated^6^ is included as the standard AKH. We call a peptide a “true AKH”, when it is 8, 9, or 10 residues long and has a pQ group in position 1; an aliphatic or aromatic amino acid residue in position 2; FS, FT, or YS residues in positions 4 and 5; a W residue in position 8; and either a Wamide, WGamide, or WGXamide C terminus.These hallmarks would also include the neuropeptides AKH/corazonin-related peptides (ACPs). However these ACPs have RD residues at positions 6 and 7, which are absent in the AKHs^13^. We assign a peptide as “AKH-like” when it is 10 amino acid residues long and has a WXGamide or WXPamide C terminus instead of a WGXamide. “Proto-AKH” peptides are longer than 10 amino acid residues, resemble AKHs, but have only 2–4 of the above mentioned AKH hallmarks.
