# Supplementary material for: Adipokinetic hormones and their G protein-coupled receptors emerged in Lophotrochozoa
Source: Sci Rep. 2016 Sep 15;6:32789. doi: 10.1038/srep32789 (PMC5024129; doi:10.1038/srep32789)
Supplement: Supplementary Information [file srep32789-s1.pdf]

Supplementary Information

Adipokinetic hormones and their G protein-coupled receptors emerged in Lophotrochozoa

Shizhong Li, Frank Hauser, Signe K. Skadborg, Stine V. Nielsen, Nikolaj Kirketerp-Møller, Cornelis J. P. Grimmelikhuijzen

Table **S1.** Primer sets used for cloning of *C. gigas* AKH and the four *C. gigas* AKH receptors.

Figure S1. cDNA and corresponding amino acid sequence of the *C. gigas* AKH preprohormone. The signal sequence (highlighted in grey and underlined) is followed by the AKH sequence (highlighted in yellow). The KR sequence (highlighted in green) is recognized by prohormone convertase that cleaves the protein C-terminally of the R residue. Subsequently the C-terminal KR residues are removed by a carboxypeptidase, after which the G residue (highlighted in blue) is converted into a C-terminal amide group. The C-terminal half of the preprohormone is highlighted in grey and contains two C residues (underlined) that form a cystine bridge. The cloned cDNA sequence was submitted to GenBank with the following accession number: KM205073.

Figure S2. Dose-response curves of the activation of *C. gigas* AKHR1-A by various peptides. (A) *C. gigas* AKH (Cg-AKH). **(B)** *Locusta migratoria* ACP (Lom-ACP). **(C)** *Nasonia vitripennis* ACP (Nv-ACP). **(D)** *Tribolium castaneum* ACP (Tc-ACP). The E_{50} values are given in the boxes belonging to each panel. All other details are given in the legends of Fig. 5.

Figure S3. Alignment of the amino acid sequences of *C. gigas* AKH, the three insect ACPs that were tested in Supplementary Fig. S2 online, and *C. gigas* corazonin. Note that the AKHs and ACPs are all 10 amino acid residues long, while corazonin is 11 residues long, which might explain why corazonin does not activate the *C. gigas* AKHR1-A and -B receptors (Fig. 5). AKHs, ACPs, and corazonins have many residues in common, but the ACPs have RD or KG residues at positions 8 and 7 that do not occur at these positions in AKHs.

Figure S4. Amino acid sequences and GenBank accession numbers of the preprohormones of the peptides given in Table 1. The signal peptides are underlined. The biologically active sequences are highlighted in yellow, the dibasic cleavage sites for prohormone convertase are highlighted in green, and the glycine residues, which are converted into C-terminal amide groups are highlighted in blue. Cysteine residues that could form cystine bridges are highlighted in red.

Figure S5. Amino acid sequences and GenBank accession numbers of the receptors used in Fig. 8.

Figure S6. Alignment of the peptides given in Table 1 plus selected AKHs, ACPs, corazonins and GnRHs. It can be seen that the proto-AKHs are more similar to AKHs (and ACPs) than to GnRHs.

Figure S7. Phylogenetic tree of the peptides aligned in Supplementary Fig. S6 online. Again, it can be seen that the proto-AKHs are more related to AKH (and ACP) than to GnRH.

Table S1. Primers used for cloning of *C.gigas* AKH, *C. gigas* AKHR1-A, *C. gigas* AKHR1-B, *C. gigas* AKHR1-C, *C. gigas* AKHR1-D, and *C. gigas* AKHR2 cDNAs. The sense primers for cloning *C. gigas* AKHR1-A and *C. gigas* AKHR1-B contain a *Pst*I restriction site marked with green, while all the antisense primers for cloning the receptors contain a *Sac*II restriction site marked with red. A Kozak consensus sequence marked with blue was included in the 5' end of the sense primers, which began at the first nucleotide of the receptor coding DNA sequence to facilitate the gene expression in CHO cells.

Gene	Sense 5'-3'	Antisense 5'-3'
C. gigas AKH	ATGCTCTGCAAATTCTGTATTCT	CCTCCTAATGTTAAAGATTTTGAGT
C. gigas AKHR1-A	CTGCAGCCACCATGAATTCCGACGCGGAT	CCGCGGTTCAGTGCTTGTCCATCGT
C. gigas AKHR1-B	CTGCAGCCACCATGAATTCCGACGCGGAT	CCGCGGTCTTAATTAGTGTTGCTATAGC
C. gigas AKHR1-C	GCCACCATGGATGAACATCTTTCGACT	CCGCGGTCTTAATTAGTGTTGCTATAGC
C. gigas AKHR1-D	GCCACCATGGATGAACATCTTTCGACT	CCGCGGTTCAGTGCTTGTCCATCGT
C. gigas AKHR2	GCCACCATGAATTTAACATATAGCTAT	CCGCGGCTACTTGAAATTGTCTTGTT

1 ATG CTC TGC AAA TTC TGT ATT CTG GCT GTA GTA GCA CTG AGC CTT CTC TCG TTG ACG CTT

M L C K F C I L A V V A L S L L S L T L

61 GGA CAA GTT AGC TTT TCC ACA AAC TGG GGC AGT GGA AAG CGT TCT TCA TCT TCA TTC ATA

G **Q V S F S T N W G S** G K R S S S S F I

121 GCT CCT CAA GCT GAC GAT GCC TGC TGG ACG AAA TCA AAC GCC AAA CTA CTG TAC GAC TTA

A P Q A D D A **C** W T K S N A K L L Y D L

181 ATG CAG GTG ATC CAG AAA CAA GTT GAT CGT CTT GTT GCT TGT CAA AAC AGT AAC GAC GAT

M Q V I Q K Q V D R L V A **C** Q N S N D D

241 TTG CAT CGG ATA TGA CTCAAAATCTTTAACATTAGGAGG

L H R I *

Fig. S1

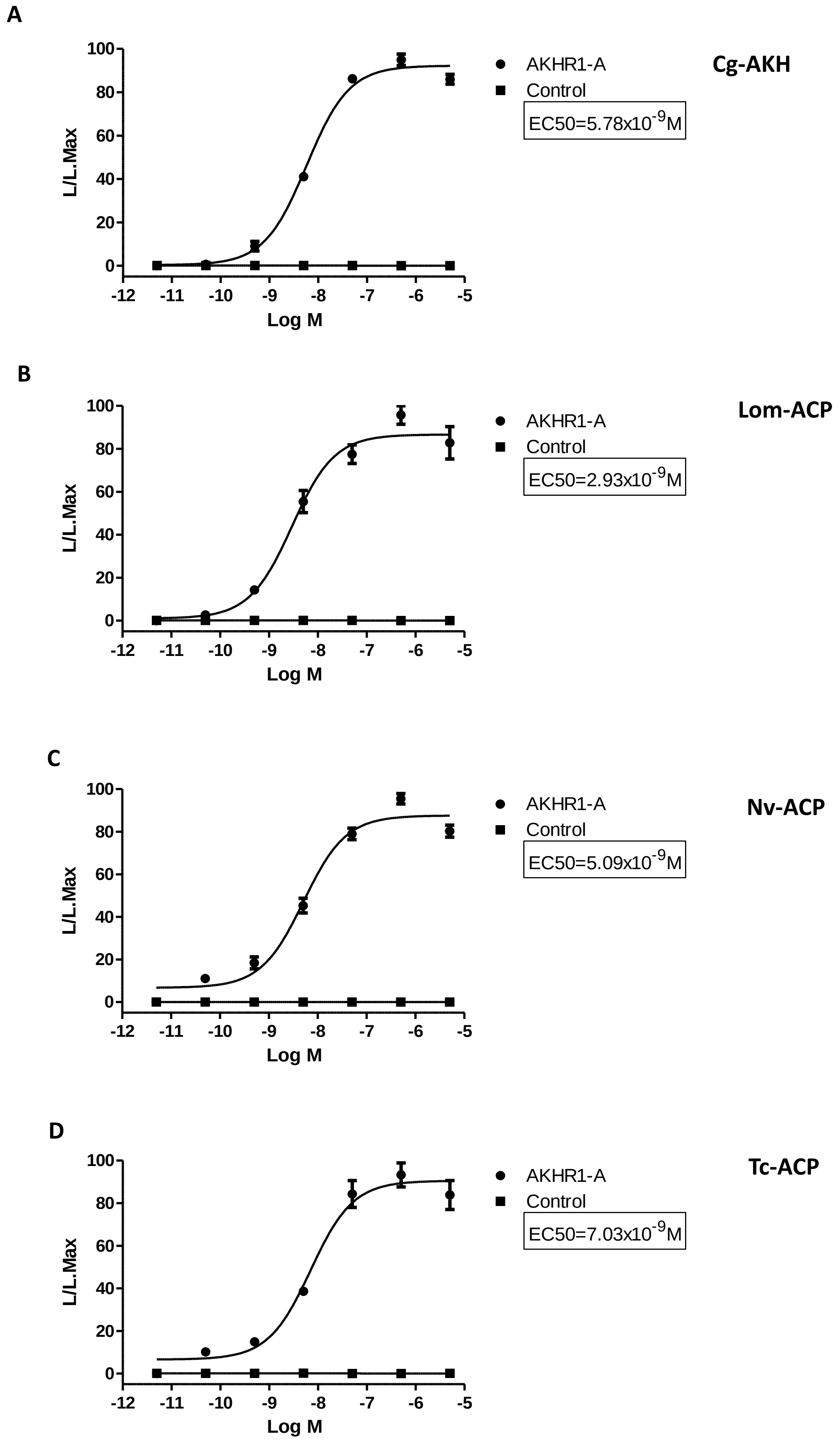
A
Cg-AKH
B
Lom-ACP
C
Nv-ACP
D
Tc-ACP
AKHR1-A
Control
EC50=5.78x10-9M
EC50=2.93x10-9M
EC50=5.09x10-9M
EC50=7.03x10-9M
L/L.Max
Log M

Fig S2

Cgig-AKH **pQ-V**S**FS**TN**W**GS**amide**
Lmig-ACP **pQ-V**T**FS**RD**W**SP**amide**
Nvit-ACP **pQ-V**T**FS**KG**W**GP**amide**
Tcas-ACP **pQ-V**T**FS**RD**W**NP**amide**
Cgig-CRZ **pQ**NYH**FS**NG**W**QP**amide**

Fig. S3

Fig. S4

Locusta migratoria AKH-1 (P55319)
MVQRCALVVLLVVAVAAALCSAQLNFTPNWGTGKRDAADFADPYSFLYRLIQAEARKMSGCSN

Globodera rostochiensis AKH (from EST, EE266432.1)
MPSLVFPTLCFFVSAVVLANSQMTFSDGWGKRSAVAPLALLADRHMTEPTKRQQKLKPFANLFVSP
DEDTLTMNPEMEACHAGYAQRLIQLHEQMLSLFSTYQQCQTKAATETKKAMRQLR

Hysibius dujardini AKH (from EST, CF075783.1)
MGSAVQSLGLGFLGALLLVQVLFFQVPTANSQLSFSTGWGHGKRSGNVPVNLAPADYRNGKANGDL
CSAAYNEMAVARIQELIQEEGLRQLACRKR

Priapulus caudatus AKH (from WGS, AXZU01076239.1)
MHCILILATCLLGLYIHESSAQIFFSKGWRGGKRQEAPLMRHPTDVEEDTNQLVTTALGKESLKLG
PSTEPLVDVSLRDAKAWHLTYRTPS

Aplysia californica AKH (XP_012941224.1)
MESSSILLILVVLVIGTSTCLAQIHFSPDWGTGKRAVSTVTEKEIPHCWQIADKEIIDIMLLIQRT
AKKLSSCLNTCPEL

Crasssostrea gigas AKH (KM205073)
MLCKFCILAVVAVSLLSLTLGQVSFSTNWGSGKRSSSSFIAPQADDACWTKSNAKLLYDLMQVIQK
QVDRLVACQNSNDDLHRIW

Lottia gigantea AKH (from WGS, AMQO01003719.1)
MSLSRNLSVLVCLCCLLSMCLAQIHFSPTWGSGKRSAPVQTYPDSSNTNCYDKLNTKILYQLVKII
KKESEELTACLLQDDDFLRR

Bithynia siamensis goniomphalos AKH (from TSA, GAQQ01002646.1)
MAQTTMTSLLAVLLLLSLVLTPVTSQIHFTPGWGSGKRSGRDDPSSSSSSSASSSSHSSSSIPLPF
SSIDSCWAEADFRLFLQLARLVKREAKRFSQCLETENSMAENQENGPDFL

Tritonia diomedea AKH-like (from EST, EV287376.1)
MKIPDMHRTLHCSIPVLVLLMCVCSSLAQIHFSPGWEPGKRSMEEPTRANKMACYDQLDMSLLLDI
LKIIKRQAEKLSYCTKSCPQL

Hyriopsis cumingii AKH (from EST, GW693635.1)
MQTTCIIAVTLVALWTFTSAQISFSTNWGSGKRSGYQDLQSAACLDDLEINVMKDLAALIKMEANR
AAKCIIDAKTRKLTGVYETPDK

Mytilus galloprovincialis AKH (from WGS, APJB010624986.1)
MKSFFLICLLISLIGDSAEQISFSTDWGSGKRSFLESYENICKSKLEQSLIFDIASLVQVNICPSL
GKDSLAF

Biomphalaria glabrata proto-AKH (from WGS, APKA01093331.1)
MQNARFVTLLTLMTIGWTLGQIHFTPDWGNNGKRSLTSQFEEESSYSSISCLEQTDLKFLLDIAKL
LTKPEQTQKEPCDLKLMKTKDLEVVYK

Capitella teleta GnRH-2 (from WGS, AMQN01004296.1)
MKHFTLLLVACIVVAYHMNTASAQFSFSLPGKWGNGKRAFSFSLPGRWGAQGKRASGWTGATTDCS
RMESDGMMSVYAAIQEEAIKMLECMGKTSADKQDAS

Capitella teleta GnRH-3 (from WGS, AMQN01001996.1)
MVVLVACSVLAFQLHSAEAQGFSFSLPGKWGGAGKRSGGVPWLKDGLGECGTYDPSLVVELYKIIQ
AEAFRLNECLMKAQREGN

Capitella teleta proto-AKH (from WGS, AMQN01001098.1)
MSCLIRVVIVLFGLSLLVLCCDGQLTQSLGWGSAGSPGKRNFRSYEAAGYNDQLMSSTECRMSAQT
YRVMTDMLEREISRLQRCR

Platynereis dumerilii proto-AKH-1 (from EST, JZ447807.1)
MRAWLVLLVACIVIAYQAHTSDAQFSFSLPGKWGNGKRAALGWGKRGECGDFDPDAIFNVYRAIQA
EALRINECMQQKLEEESKKH

Platynereis dumerilii proto-AKH-2 (from EST, JZ459150.1)
MMRLWVVYLTVCVMMLFAFFPHVCHSQLTQSLGWGSAGSSGKRSVKSPIYYGDDDDYDYDVRQVRK
NRLTLSEALCSQDEKDAISKIKKLIQREVVRQRYCNRKK

Helobdella robusta GnRH (from WGS, AMQM01007250.1)
MNCLALFLACLVLSTVIILPAKSQFSFTPPGKWPFGTGKRDKIISDDDLTSFQEECFKQDPRILSQ
TFLRLQKEANIAKECLKKIYPNARK

Lingula anatina proto-AKH (from WGS, LFEI01000562.1)
MVTKHNMLWISVTVIVMVLQNTNCQWHQTLGWGAAGMVGKRTNPSSIFSQDSQDDSLCDDRRNSEV
MKLIRDLLQVSDRCWSFLCRLSGSCHTRLSCRG

Fig. S5. Amino acid sequences and GenBank accession numbers of the receptors used in Fig. 8.

>Acyrthosiphon_pisum_AKHR (XP_003245941.1)
MEVMDSDANTVLHSVTKGVHVGPPPVWPGMDNVTNASMFDESNLPYDMKFNEGHVVAIVTYSILMV
VSAIGNITVLTIILKRRRKAGTRIHAMLMHLAIADLLVTFLMMPLEITWAWTVQWVLGDPLCRIMS
FFRIFGLYLSSFILICISVDRYLAVLQPMRLYQMDRRGKLMIAVAWIASVVCSLPQTYIFHVERHP
NATWYEQCVTYNAFSSKLHELAYLYFGMFMMYWLPLIVILFCYASIIIEIYRRSRESICDNVRRLG
FLGRAKSRTLKMTIIIVIVFVVCWTPYYIMAIWYWTDHKSAQMVDQKVQHALFMFACTNSCMNPIV
YGAFNIRTRRTLVTQVRPSASLASTACTTEIRLPALSISVRRVE

>Aedes_aegypti_AKHR (XP_001655248)
MSNAILKTERDEVLNYSHSYGENYNNDVNTMPYVLSSSTSKTGVFDNETWYGTNSSNWNEPLPIDM
QFNDGHKLQIVVYSVLMVISAIGNITVLALLIKRRLKSHSRIDMMLTHLAIADLLVTFLMMPLEIG
WAATVQWRAGDIMCRVMAFFRTFGLHLSSFVLVCISVDRYYAVLQPLNLSKSRGKIMILIAWAMAT
LCSAPQPFIFHVEIHPNHTWYEQCVTYNTFSNDNYHTVYNILVMMFMYALPLLTIICSYASIYMEI
FRHSRMPNSEGFRRSSIDVLGRAKRRTLKMTITIVMAFVICWTPYYVMSVWYWLDQKSAENVDQRV
QKGLFLFACTNSCMNPIVYGIYNVKLRKKKKPDGVKSGQSSVILRNSAKYTRHSESIRSSSGKIRG
YRDLDIPSCAIKPV

>Anopheles_gambiae_AKHR (ABD60146.1)
MPNTMAAHINQRIEDHRNLADWSYYANETAGEEYYEMPIDMRFNSGHILSIMVYTTLMVFSATGNL
TVLSILAQRKVRASSRINIMLAHLAIADLLVTFLMMPLEIGWAYTVRWTAGDLMCRVMAFFRTFGL
YLSSFILICISVDRYFAVLKPLKVHEHRAVLMIAAAWIMSGLCSLPQAFIFHLEGHPNITGYQQCV
TYHYFEEEIYQIIYNVLVMCLMYTFPLIVILYCYGSIYYEIFSRTNPRNLESFRRSSIDVLGRAKR
KTLRMTIMIVIVFVVCWTPYYVMSLWYWLDKESAKNVDQRIQKGLFLFASTNSCMNPVVYGVFNVR
KKHTKKLLKTTHEKSCGSHLTMRA

>Apis_mellifera_AKHR (NP_001035354.1)
MESSIKIITTTGLENWRVNNSNYTELLPIDMRFNEGHIVSIVFYSVLMIISAIGNTTVLILITCRK
RVSKSRIHIMLMHLAIADLLVTFLMMPLEIGWAITVSWKAGDVMCRIMAFFRMFGLYLSSFVLVCI
SMDRYYAVIKPLQLWDVDKRGKIMLSFAWIGSVVCSLPQTIVFHLETHPNVTWYSQCVTFNAFPTY
THEITYSLFGMIMMYWFPLVVIIYTYTSILLEIRRRSKKSEDDKIRRSSIGFLTRAKIRTLKMTVI
IIAVFFICWTPYYVMSLWYWIDRNSAYKIDQRIQKGLFLFACTNSCMNPIVYGAFNIRDRNKTSAR
PTTIETRVTPLSLSLKLLD

>Aplysia_californica_AKHR (XP_012941361.1)
MPETHSCITSQKVLTKFTTTVSLIAMLFPAAMALATKHLHPPMTARLQEDPLSLQSPSALRSTVSG
TENASSLDHTYEVQVVVEREFSAKSGREDEINALNTAAAVAATAASTTTPINNNSYSHCINLTLSE
IQTNYTECLHPLPKELYFTDDNAVSVAAYSCLFVVAAVGNLTVFITLFRNRGVKSRINMFIMHLAI
ADLIVTFMMLPLEIAWHSTVAWKAGDAVCRLLMFFRAMGFYLSSCILVSISLDRYFAIMKPLSISD
AGNRSKMMLTISWFLSIVASIPQSIIFHVERHPKYKWFEQCVTFNFFPTPEHELAYNLFSIIALYG
LPLVIITSSYCVILVRISKKSRQSKDEMRISGYDGTPAEQGGLRRSGIGNIERARSRTLKMTLVIV

GAYVLCWTPYFVISAWYYFDRTSAVKIDGKVQRGLFLFAVSNSCINPIVYGMFTAAFRRESKRWGD
WLKTTFSYRGSGPRTYCGAGASHHCAL

>Bombyx_mori_AKHR (NP_001037049.1)
MDIDEKVSGPGGASQKNWSHLLHVNNTYDELPLEMRFNYSHMVSMTVYSVLMVISATGNLTVLYQL
VRRRRAKRASRLDILLMHLAVADLMVTFLMMPLEIAWAGTVQWFAGDLMCRVMMFTRTFGLYLSSF
VLICIAVDRYYAILKPLNVTWEATVRRAIIVAWVCAGLASLPQSFIFHVEEHPEVKGYNQCVSYGS
LPTEKHEFAYFLVNMILMYVIPLVSTLYCSCAALFEIIRRANTANDKMRRSGIGLLGRARARTLKM
TVTIVLVFFTCWSPYYCYCLWYWIDKESIKNLDPALQKAMWLFSCTNSCANPIVYGVFNRNRWNWR
AGKFQNGRCRSGSGRKGSRLPHGESTEISAATLSRARHSNGSDHNGRRDSSYANQNGPQKHWNTIN
NNHVTNGMV

>Caenorhabditis_elegans_AKHR (NP_001249720.1)
MTTINCSRSVPPDVTVNDSVLSIVFTYLALFILAFVGNVTMFLILCRNQLVKVRRVHSLLLHMNIA
HLLVTLVVMPKEILHNYMVAWFAGDVMCRICKFFDVFAISLSMNVLICITLDRFYSIFFPLYAMRA
RKSVQRMVSFAWTISFVTSAPQLYLFKTATHPCFDWYTQCVSKNFIGELSNDVVFFFSIVNIIQVY
IAPLFVTVVCYSLILWRISRKSKLVGEKESEKSSELLLRRNGQNNLEKAKSRTLKMTFVIVLAFIF
CWTPYSILMFLHFLRHTDWIPKDIRKFIYAFAVLNSAISPYLYGYFSFDIRKELQLLFACSKATAA
DRHLSCSANVSRNQVTERMRKRSASACNFDGGKTNNTLSPRPPRGHSLRHKPSSSGIDKRNHNVQL
EIIDF

>Capitella_teleta_AKHR1 (ELU01220.1)
MNFTTSLDVWQQENASTVGPGAAGPPREIQFNGDSVVSIVTYSVLFCFSAVGNLSVFLTLVAARYQ
RRASRVSLFILHLTVADLFVTFVMIPMEIGWHVTVSWKAGDVACRLLMVLRTFGFYLSSLILIAIS
LDRYLSIAHPISIQSAGRRSRIMILVAWVIGFIASLPQSLIFHAAQHPEFELYVQCVTFGSFPTQA
HELAYNLFNMFLLYSIPLIAIVLCYSLILREMDASQNTFEIMADPDPIVGQTGRLDKARLHTLKMT
IVIVAVFIVCWTPYFIMHILWWIDKNTAEKVNPKIQRGLFIFAVSNSCVNPVIYGQYFM

>Capitella_teleta_AKHR2 (ELT92261.1)
MDCSEALIACGDPYHGLPLNETANTSDSNFEVPRELLFNTDAIVSVSILGVMFLVAAVGNLTVFAI
LFFNRHSRLSRVSIFIMHLSISDLIITFIMIPMEIGWHVTVAWKAGDVGCRVLLFFRAFGFYLGSF
ILVAISLDRYLSITKPLSLVDAGKRGRIMLRFSWLFAFVAAIPQSIIFHVEQHPIHTWFSQCVTFN
FFPSDQHEMAYNVSTVCMMYVVPLLLITACYSLILREVSKKTSSTRTVSGGQCRVRMSGNGRLKMA
KARTNTLRMTIVIVLVFVICWSPYFCMLLVWWIDRGQAVKVPPKLQRAIFIFAVSNSCVNPFVYGT
CSHIYINGASMSTSMKPRYSNVCLN

>Capitella_teleta_AKHR3 (ELU00909.1)
MEYMNNQSELDPTSWTVEMDNYTAASIPRDMVFGEEGLISVIAYCILFLIAACGNLTVFITLYRNR
HRKSRVNLFIMHLSVADLIVTFIVLPLEVTWHITVAWYAGDAACRIIMFFRAMGFYLSSFILIAIS
LDRYFAILHPLSLNDADKRSKLMIIFSWAFSVIASIPQSIIFHVEQNPTYSWFSQCVTFNFFPSEG
HELAYNMFNMITVYALPLVVIIVSYSLILCEMTRKTRESQEDVKAPSKMNASERLRRSNVGHIQRA
RIRTLRLTIMMVLVFILCWTPYFVMSSWWWFDRESAAKVDPRIQKGLFLFAVSNSCMDPIVYG

>Capitella_teleta_AKHR4 (ELT96363.1)
MGSPTNSCDTCAPDLMTVAPSEVPSELTGSANNLLSITAYSVLMVIAACGNLTVFITLFRNRHRKS

RVNLFIMHLSIADLVVTFIMLPLEIAWHTTVAWIAGDVACRILMFWRTFGFYLSSFILIAISLDRY
FAITRPMSLSDADRRGKLMLILAWLFSIVASSPQSVIYHVESHPNHPWFKQCVTFNFFPTTTHQMI
YNVFCFSALYLAPLVVIVISYSFILIEITKRSREGRGGPDHVKRKQSTNSMIASTGSLRRSGVGRM
EKARIRTLKMTIVIVFVFVMCWTPYYAMIIWHWVDKTSAEQVDEKIQRILYLFAVSNSCMNPIVYG
VVHHPTKEKDAIRVSLAALCLFDPFAAQSTFQDNDQ

>Capitella_teleta_AKHR5 (from WGS, from AMQN01016278.1)
MGMEPCAAASKLECHSDGTTILPVQVVTTDPDITFNDERALAVAGYSILFAIAALGNLTVFITLIR
NRRKRTAYSRLNLFIMHLCIADLIVTFVMMPLEIVWHVTVAWLAGDVACRLLMFWRAFGFYLSSFI
LVAISLDRYFAIARPLSLADAHSRGRLMLVLAWALSCVASIPQSVIFHIERHPKYPWFSQCVTFGF
FPSPRHELAYNLFNISAIYGFPLLVIVFSYSAILRHMSQRNASQHLRNRLQKSKLYSSTVSVFFIC
WTPYFVISAWWWFHHSSADKIHPGVQKGLFLFAVSNSCMNPVVYGMFSTVCRRTLSRLRKQAYDYL
SLCMATPEE

>Crassostrea_gigas_AKHR1-A (KM205066)
MNSDADADTTIRFNQSSLYEDVYSTAPNDGVLLMKFTCENGFDEFQKNGSCQNNTTEMLPMDLVFT
DENMVTIVAYTCMFIVAACGNLTVFITLFRNRNIKSRVNQFIFHLSIADLVVTFIMLPLEIIWNIT
VAWKAGDPACRILMFFRILGLYLSSFILVTISLDRYFAIVHPLSLNDADKRGRIMLILAWCFSIVA
SIPQSVIFHVETHPKYRTFRQCVTFNFFPSHNHELAYNLFNLITLYALPLLIITTSYSLILWEISK
KTKQCKEETKCLSTRSRLRRSSVGNMERARIRTLKMTLVIVSVFVICWTPYFVLSAWWFDSDSASQ
LDPKVQRGLFLFAVSNSCMDPIVYGMFTINFKREFVRCCCCLKTSWKRHKLQRLTGKFQTSTGVQR
GPLSHTASNNSCRSLPGTNVVKFFDDAAICGNGGSPVLSRDLLKPKLDTGSRRANVNNFMKVNLPA
LEKSVTMDKH

>Crassostrea_gigas_AKHR1-B (KM205067)
MNSDADADTTIRFNQSSLYEDVYSTAPNDGVLLMKFTCENGFDEFQKNGSCQNNTTEMLPMDLVFT
DENMVTIVAYTCMFIVAACGNLTVFITLFRNRNIKSRVNQFIFHLSIADLVVTFIMLPLEIIWNIT
VAWKAGDPACRILMFFRILGLYLSSFILVTISLDRYFAIVHPLSLNDADKRGRIMLILAWCFSIVA
SIPQSVIFHVETHPKYRTFRQCVTFNFFPSHNHELAYNLFNLITLYALPLLIITTSYSLILWEISK
KTKQCKEETKCLSTRSRLRRSSVGNMERARIRTLKMTLVIVSVFVICWTPYFVLSAWWFDSDSASQ
LDPKVQRGLFLFAVSNSCMDPIVYGMFTTTFRRETQKWGRWL

>Crassostrea_gigas_AKHR1-C (KM205068)
MDEHLSTNESTSLPSYLKFSDNYAHSIISLGVVFLIGALGNIASFVTLLCSKNRKSPTNFFLLHLS
SADLLVMFVIPVGEMIHNATIAWLGGNFLCKLYHFLWNFGQYVATFLLCCISIDRYLAFVFPLRSL
TQSPRTRSFMAATAWVLSAILSAPESVIFHVETHPKYRTFRQCVTFNFFPSHNHELAYNLFNLITL
YALPLLIITTSYSLILWEISKKTKQCKEETKCLSTRSRLRRSSVGNMERARIRTLKMTLVIVSVFV
ICWTPYFVLSAWWWFDSDSASQLDPKVQRGLFLFAVSNSCMDPIVYGMFTTTFRRETQKWGRWL

>Crassostrea_gigas_AKHR1-D (KM205069)
MDEHLSTNESTSLPSYLKFSDNYAHSIISLGVVFLIGALGNIASFVTLLCSKNRKSPTNFFLLHLS
SADLLVMFVIPVGEMIHNATIAWLGGNFLCKLYHFLWNFGQYVATFLLCCISIDRYLAFVFPLRSL
TQSPRTRSFMAATAWVLSAILSAPESVIFHVETHPKYRTFRQCVTFNFFPSHNHELAYNLFNLITL
YALPLLIITTSYSLILWEISKKTKQCKEETKCLSTRSRLRRSSVGNMERARIRTLKMTLVIVSVFV

ICWTPYFVLSAWWWFDSDSASQLDPKVQRGLFLFAVSNSCMDPIVYGMFTINFKREFVRCCCCLKT
SWKRHKLQRLTGKFQTSTGVQRGPLSHTASNNSCRSLPGTNVVKFFDDAAICGNGGSPVLSRDLLK
PKLDTGSRRANVNNFMKVNLPALEKSVTMDKH

> Crassostrea_gigas_AKHR2 (KM205070)
MNLTYSYQFTDLSEVEKVETTMKIMNTTVGTGSGNMSDKGLPREMTFNDDNVMSVIAYACLFLFAA
SGNLTVLVTLLKSRRYKSRVNTFIMHLSIADLIVAFIMLPLETAWHVTVAWEAGDAACRILMFFRA
LGFYLSSFVLVAISLDRYFSIVHPLSIHDADRRGKIMLTMAWLLSIIASLPQSIIFHVERHPLFTW
FEQCVTFNFFPTEQHELAYNLFNVITVYLLPLVIITTSYSLILYKVSKTARRDEEERREFGLYTHR
SHTYPLSRSGLISCTRAGIIGKARIRTLKMTLVIVTVFVLCWTPYFLMVSWFWIDRESAKNIDTKV
QRGLFIFAVSSACLDPIVYGMFTAAFRKEARRWLGWFKDRLNRMGIYTMTPNTASEQDNFK

>Culex_pipiens_AKHR (from WGS, AAWU01041683.1)
VTFLMMPLEIAWAWTVQWVAGDIMCRLMAFFRTFGLYLSSYVLVCISVDRYFAVLQPLKLSKARGK
IMIIGAWMFSTVCSSPQALVFHVEPHPKVSWFLQCVTYNSWNGNYQFIYNILGMVFMYALPLLTII
CSYASIYMEIFRHSRMPNSDGFRRSSIDVLGRAKRRTLKMTITIVTVFAICWTPYYVMSVWYWLDA
SSAYSVDQRVQKGLFLFACTNSCMNPIVYGV

>Daphnia_pulex_AKHR (ACD75498)
MCSNDSSSLSGNMCMMTEDASFSGESTVNPEFDSASSISGGSSSSTTTTVDLSMLPIDMTFNDGHV
VSIATYSVLLIISVCGNITVLVNLIKRRHISNPRVNIMLTHLAIADLLVTLLLMPIEIGWAATVQW
RAGDFSCRMLAFFRTFGLFLSSFVLVCISIDRFGAILQPMKLDYWKRRGRFMLAIAWACSVICSLP
QVFVFHVKAHPEYPWYEQCVTFDSFPTKAHEISYAAFGMMMVYVLPLAVFVFTYSSILCEISRRSK
EAVGQEEGIRRVTVGTLGRARIKTVKMTLVIISVFIFCWTPYNIMSIWFWCDRDSALQVDQRIQKG
LFLFACTNSCFNPMVYGYFSRRTVRRSHHELHRKVVYHPSRMASRALGPSLRVDCNSKSLEGAATE
PCLPQQHLPVATADGPSCQGQAEPIFTASSIAVAVKRSNSWLNNRSNPTVVTHIF

>Drosophila_melanogaster_AKHR (AAC61523.1)
MAKVAEENDHRDLSNWSNVNDTNGTIHLTKDMVFNDGHRLSITVYSILFVISTIGNSTVLYLLTKR
RLRGPLRIDIMLMHLAIADLMVTLLLMPMEIVWAWTVQWLSTDLMCRLMSFFRVFGLYLSSYVMVC
ISLDRYFAILKPLKRSYNRGRIMLACAWLGSVVCSIPQAFLFHLEEHPAVTGYFQCVIFNSFRSDF
DEKLYQAASMCSMYAFPLIMFIYCYGAIYLEIYRKSQRVLKDVIAERFRRSNDDVLSRAKKRTLKM
TITIVIVFIICWTPYYTISMWYWLDKHSAGKINPLLRKALFIFASTNSCMNPLVYGLYNIRGRMNN
NNPSVNNRHTSLSNRLDSSNQLMQKQLTNNSLLNGRGQVMAAAVSATTKLANVVSLKGTANGNGSA
AAAGTVPITPPLTVTIAPLATDDEANDDSCLSAVTIRCQDQSPIRQK

>Helobdella_robusta_AKHR (ESN91517.1)
NLTVIITLTLCKKRQRRTRVEWFILNLAIADIFVALYNNPIEIAWAVTISWNAGDAACKILMFCRA
FGFYLSSFILVAISFDRYFAVAKPLSTGTADERGKIMLITAWTTAVFASLTQVFFYLAKHPNLENF
YQCVVTNDSFPNHIVEMFYHAFNFISVYFLPLVVIIVSYSLIIIIISKRSRQTIGCNSNRVTLRIS
GLNRIEQARAKTLKMTIVIVLAFIICWTPYYVLAAWCWLFQDSVQNYVNQWIQEILFIFAISNSCA
DPIIYGLL

>Lottia_gigantea_AKHR1 (from WGS, AMQO01002634.1
MIVPKLISMMPDDSMGTSKLYRKTSEENENTTMGAYSTNITLPREMTFNDDNLVSVIAYSCLFVIA

AAGNLTVFITLLRNRGFKSRVNSFIMHLSVADLIVAFIMLPMETAWHITVSWKAGDFTCRLMMFFR
AFGFYLSSFILVTISLDRYFSIVHPLSIHDAEKRGKIMLTIAWILSVVASLPQ

>Lottia_gigantea_AKHR2 (ESP00340)
MAENQTDIRCNDSLLNVTPCNTFPSLPRELTFNDDSLVSVITCSCLFIIAACGNLTVFITLFRNRN
IKSRVNLFIMHLSIADLIVTFVMLPLEAAWHLTVAWKGGDMACRAMMFFRAFGFYLSSFILVTISL
DRYFAIMHPLSLNDADRRGRIMLSLAWICSIVASIPQSVIFHVERHPVYEWFTQCVTFNFFPSPSH
ELAYNLFNVIAVYGLPLVIITGTYSLILCEISKKSRQSQMQLESSSIRQKGELRRSAMGIIERARI
RTLKMTLVIGKIHIVFFLLFLSGSLVD

>Nasonia_vitripessnis_AKHR (NP_001161243.1)
MTTAPVNATTVASLDYDDLPIDMRFNAGHVVSIVTYSILMIISAVGNITVLALLLRRRGNAARTRI
NTMLIHLAIADLLVTFLMMPLEIGWAATVSWKAGDAMCRIMSFFRMFGLYLSSFILICISVDRYHA
VLRPLQMIDIDRRGRFMIAGSWICSALCSAPQMVVFHVEAHPTFTWYEQCITFNTFPSFTHELTYS
LFGMVMMYWFPLIVIIYTYTSILAEMYRRSKDTTSDRIRRSSLGFLGRARVRTLKMTIIIVLVFFI
CWTPYYVMSLWYWIDSVTATKVDLRIQKALFLFACTNSCMNPIVYGAFNIRKGNKAPVRAPTIETR
VTPLSLSIRLLA

>Tribolium_castaneum_AKHR (NP_001076809)
MNFSETLWKMKDPMSASETVQDHRNLLDWSKTSLDNATEHKLPISMRFNEGHQLSIIVYSILMVFS
AIANTTVLVLIVKRRRKTPSRINTMLMHLAIADLLVTFLMMPLEIGWASTVSWYAGDAMCRIMMFF
RMFGLYLSSFILVCISVDRFYAVLKPLYLRALDRRDKFMLLGAWLGATLCSIPQMVVFHVESHPNI
TWYQQCVTYNVFPTYAHELTYLLFGMVMMYALPLAVIIFSYASILLEIRRRTRNPYGDSVTRSSLA
FLGKAKVRTLKMTIIIVLVFFVCWTPYYVMCIWYWLDRESAKNVDQRIQKALFLFACTNSCMNPVV
YGVFNIRARRTGRKVSPRVNTIKHTSCIPTPNGDSRLPPLEISLKTLE

>Strigamia_maritima_AKHR (from WGS, AFFK01020326.1)
MSLQLSTTSSLWLQDNKSYTNDSQNQFPPELQFGEHHLVSIVTYSVLMVISGIGNLSVLVSLVFRR
RKKKSLVDLMLIHLAVGDLLVTFVMMPLEIAWAATVSWWAGDAMCRIFAFFRTFGLFLSSFVLVCI
SIDRYFAVLHPMAIYYSYRRGKLMLIGAWIMSTICSIPQIIVFHVERHGQITWFEQCVTFNSFPSR
IYELAYYMFGMIAMYGLPLVVIIVSYGAILIEISMKSHEYKEQVQSSALGECRLRRSGFGNLSRAR
TKTLKMTVLIVAVFVMCWTPYYVMCLWYWFAPESAKQVDPRIQKALFLFASSNSCMNPIVYGMFSF
NCTLTLRPSTREEKRSKYLDSHRWSERTVVTNNLLDVESQDDPNGTCIAKVVTFPASMSTKESQDS
D

>Aedes_aegypti_ACPR (XP_001653920)
MIRGHTETTVAVIIVYCVLFIIAAGGNLSVVITLFRSRRHRRSRVSLMICHLAVADLMVAFIMIPL
EVGWRITVQWHAGNVACKVFLFMRAFCLYLSSNVLVCVSLDRCFAVIYPLRVSAARKRGKIMLGGA
WFIAFANAIPQSIIFRVQHHPNVPDFTQCVTFGFFTTPAMETAYNLFCVVAMYFMPLMVISAAYTV
ILCEISNRSREKETSDTSHTGGMRLRCNDLTHIERARQRTLRLTITIVVVFVWCWTPYVVMTLWYM
FDRESALKVDGAIQDGLFLMAVSNSCMNPLVYGSYAMKCRRPWRRQMAPNGVQTPNAAQRRSTGK

>Anopheles_gambiae_ACPR (ABX52399.1)
MYLAAGLLNIMDISLQHEYLQEYLQSAAAMANFSGANPYGLGGFGGLAPNGTGLLGGLDKNGTEVT
ITAPGHTDSTVAVIIVYCVLFVIAAGGNLSVVITLFRSRRHRRSRVSLMICHLAVADLMVAFIMIP

LEVGWRITVQWHAGNVACKVFLFMRAFCLYLSSNVLVCVSLDRCFAVIYPLRVSAARKRGKIMLGG
AWFIAFVNAMPQSIIFRVQQHPQVPGFTQCVTFGFFATPGLETAYNLFCVIAMYFLPLMIISGAYT
VILCEISNRSREKETSDSNSTGTMRLRCNDLTHIERARQRTLRLTITIVVVFVWCWTPYVVMTLWY
MFDRESAAKVDVAVQDGLFLMAVSNSCMNPLVYGSYAMKCRLPCRRRNTLGGAQTPNAAQRRSTGT
YGWWWDCMRVHVLCFPIEQTLEFPRRKGRGHGSRPSFWKLYVCSDYNSLFWKRVSMPTAMGSKNEP
SVLHEYLEKHLI

>Bombyx_mori_ACPR1 (NP_001127726.1)
MDESTQMDVTACNDTTCSDTTSTPEQNFVIGVYSILLVIGAVGNVAVLISLLRNRRRKSRVSLLMT
HLVIADMIVIFYFIPLEIGWRKTNAWLAGNVACKFLQVFRGFGLYLSSNVLVCISVDRFFAIIYPL
RLAIARKRSKMMLYVAWAFALLLSLPQSAVFRVMEHPQIPDFKQCVSFEAFSNHQQELAYNVICLS
AMYFVPLLVITICYLCIFYKISRNSKQNSEKEPPSNSRRVILRRSDQRPLVRARRRTLRMTVTIVT
VFACCWFPYATMTLWYMLDWESAMRVPKRLQDFFFIMAVSNSCMDPLVYGSYTVDLRALILALRKI
FCIRKEPTVLPGIKRPETITLVDQLRISQSRKRVRLSNPRDDLTTPRTSSEPFAYRAHHSFSERAI
IMKPTHSCDDFTLSSPKKWYSA

>Bombyx_mori_ACPR2 (NP_001127745.1)
MKMVNLNFDYEDTKEVPTSEIDKKFWEYFDATSTEISNYSSADLVPLDQGPVLATYAILLAIGGVC
NIAVLVKLAKPRRRKSRVDMLMTHLALADVCVTCGVIPLEIGWKYTNAWLGGNFLCKLLLVLRAFG
LYLSSNVLVCISIDRFFAVIYPLRLPEAKRRSRQMLYCAWVGALACSLPQSMVFRVKHHPRVIGFE
QCVSFDAFNSYEQEVAYNVFCMCAMYFLPLIVITVCYVCIFCEIRKSSKELGDKYHSGLKPVRLRR
SDRSLLERARRRTLRMTVTIVSVFALCWLPYAIMAMWYMVDRESASKVSRRIQDLLFAMAVSNSCM
NPLVYGSYTLDIRGALRRFLKKCCSSTTPEVKGQAGSSSNKNANFDTPHITEPKNIRTRLGVRFAE
TSLTAVPERLEVPRAPRGPA

>Culex_pipiens_ACPR (XP_001842868.1)
MGRKSYTDSTVAVIIVYCVLFIIAAGGNLSVVITLFRSRRHRRSRVSLMICHLAVADLMVAFIMIP
LEVGWRITVQWHAGNVACKVFLFMRAFCLYLSSNVLVCVSLDRCFAVIYPLRVSAARKRGKIMLGG
AWFIAFANALPQSIIFRVQQHPQVPGFTQCVTFGFFRTPGMETAYNLFCVIAMYFMPLMIISAAYT
VILCEISNRSREKETSDTSHTGGMRLRCNDLTHIERARQRTLRLTITIVVVFVWCWTPYVVMTLWY
MFDRESAIKVDGAIQDGLFLMAVSNSCMNPLVYGSYAMKCRWPWKRQNVPNGVATPNAAQRRSTDA
VSGMAGPHSDRLTGRDNKDELMYEKERSIKLNQFGLANGAPGQTKIGMNSTGE

>Ixodes_scapularis_ACPR1 (from WGS, ABJB010361713.1 and ABJB010787461.1)
MADNQTYHHGTHTSSGPENASSPSFPLPDDLTFNTLSLVQVIVYSVLFIAAAAGNVSVFFSLFGER
HRKSRIKLMILHLVVADLIVTFVMIPLEIIWRLTVQWTAGNVMCKAMQVLRAFGPYLSSMVLVCIS
VDRYFAVLHPFKVHDARRRGRMMLAAAWYTSLVCSSPQAFIFRVMEHPAMPGFYQCVTFAFFPSPW
HERAYNLFCLLVLYGVPLAAIMLCYGCILAEIRRSSTECAADQATDSAPARGRLRLRRSDTRHIQR
ARDRTLRLTVIIVLAFFWCWTPYVTMVLWYQFDPDGAGHVNGYLQSSLFMFAVSNSCVNPLVYGSY
TTSSKKLTDKVRGSLGRVRDGGRCLLCRRSSGKTVVSSPWKPTSSVVKSVEPDRDCCSLCSSDIHA
SDYKLDPVDGKPKSVEVSVRSDRPDKSVCEVTGGSRRGRYSV

>Ixodes_scapularis_ACPR2 (from WGS, ABJB010171383.1, ABJB010294885.1 and ABJB010610796.1)
MPHTNHSEESSEILLDVNHPNWTDPGQQLFPEELTFNTQSLVQVIVYAVLFVTAATGNIPVFFSLM
GKRHRKSRLKVMIMHLAIADLIVTFVMIPLEVGWRLTVQWTAGNVMCKVMQVLRAFGPYLSSMVLV
CISLDRYFAVLHPLKVNDAQRRGRIMLSWAWITSFLCSVPQAVIFHVMEHPQFPEFEQCVTFASFP
SSWHERLYNLFCLLALYGLPLAAIVVCYSRIFWEIHRQSRDPQVVLLLDDLSKQRRQFQGRMRLRR
SDMRHMQRARNRTLRLTIIIVLAFSWCWTPYVTMVLWYQFDPDGAEHVNGYLQSSLFMFAVSNSCV
NPLVYGSYTLTRRRFCVRCPFFGARDARTAPMTVPEVKPVVLPKDKTTVGDNSLFSSDSSVYPYEL
TIRFKGQLFSKCSKARHLPDCSCESCALSG

>Ixodes_scapularis_ACPR3 (from WGS, ABJB010083490.1, ABJB010661157.1 and ABJB011116016.1)
MSTARYGMTFGSIIEETSTSNITSQKVALAEELSFNTQSAVQVVIYCILFIVAAGGNVPVFVTLLR
NRHRKSRIKLMIMHLAIADLIVTFVMIPLEVSWRLTVQWTAGNFMCKLMQVFRAFGPYLSSMVLVC
ISVDRYFAVLHPLKVHDAQRRGKVMLAVAWYTSLVCSIPQAIIFRVMEHPAMPGFYQCVTFAFFPT
PGHEKAYNLFCLLALYGVPLAAIVTCYGRILWEIHRQSRDSHDDQSTGAESNHGRLRLRRSDVRHI
ERARNRTLRLTIVIVLAFFLCWTPYVIMVLWYQFDPEGAEHVDGYLQSSLFMFAVSNSCVNPLVYG
SYTATFEKRCCGQTTSWCWVLGNVRCKENYKLSKQPSKQTVNSWVTRTPQCQQRELQKDWCSACSS
EVYDLRSVLDKNEAGYCQSHVELTRSTTKLCRCSVDSVVLVLPSAKQVS

>Ixodes_scapularis_ACPR4 (from WGS, ABJB010179456.1 and ABJB011086824.1)
MDSVQLPSLQNGSLEDPSLDEELTFNTQSLVQVIVYSVLFVAAAAGNVSVFVSLLGKRHRKSGIKL
MILHLAVADLIVTFVMIPMEIGWRLTVQWTAGNAMCKVMQVLRAFGPYLSSMVLVCISVDRYFAVL
HPLKVHAAQQRGKKMLAVAWCTSLACSTPQAFIFRVMEHPALPGFFQCVTFASFPSPWHERAYNLF
CLLVLYGVPLSAILVCYSRILWEIHRQSREPCADQLNGFKSPGGSLRLRRSDMRQMHRARYRTLRL
TVIIVLAFFWCWTPYVTMVLWYQFDPDGAEHVNAYLQSSLFMFAVSNSSVNPLVYGSYTTGFKNLS
AKLKCCWSGGARTAQCGPSSKHTVDFWVAKR

>Ixodes_scapularis_ACPR5 (from WGS, ABJB010141465.1, ABJB010294885.1 and ABJB010934409.1)
MAIPFEAAYEFRHSINTSTEDQRGDVFHNISSPDQLLPEELTFNTQSLVQVIVYTVLFVAAAAGNV
PVFVSLLSKRHRKSRITLMILNLAVADLIVTFVMIPLEVGWRLTVQWTAGNAMCKLMQVLRAFGPY
LSSMVLVCISLDRYFAVLHPLKVNDAQRRGKIMLSWAWIISLLCSVPQAFIFHVMEHPQFQEFEQC
VTFASFPSPWHERLYNLFCLLALYGLPLAAIVICYSRIFWEIHRQSRDPQVVLLLDDVSKERQQFQ
GRMRLRRSDMRHMQRARNRTLRLTIIIVLAFFWCWTPYVTMVLWYQFDPDGAVHVNGYLQSSLFMF
AVSNSCVNPLVYGSYSSSRKGRWCRRCPFSETGISSKTTTMAPPSVHQAVAAAVKVPPTGDCSLFS
SDSSVYPYEVTIRFKGSVARTTSAGGGGPVYSCSNCSAFAG

>Nasonia_vitripennis_ACPR (NP_001164571)
MDQLQGSRMQLLQDFNNHDFRDNMSMAVPTMPPSMTFTRRTLTIIIVYCICFLVAAIGNLTVFLTL
WRGRYRKSRISLMICHLSIADLLVAFFTIPIEIGWRLTVQWIAGNYACKLFLFLRAFGLYLSNNIL
ICVSLDRYFAVLYPLRVNDARRRGKFMLSVAWFFSVLYAIPQSIVFHVENHPHHKNFTQCVTFGAF
PSDLVENTYNVFCVLTMYFIPLAIICWVYLKILCEISSKSRDNKPAGSNGTLESSNSNQGSRMRLR

RSDMSSIERARSRTLKMTIIIVVAFIFCWTPYITMNLWYVIDKKSAKEVNEMVQESLFIMAVGNSC
ANPLVYGSYAIDLKKECFRCFLPCTTTKSNADVNLIQRSLGSKFQKPEMKSPGVSKQIVHSVCQAV
HGFFKAGSGQTKSTNVCGKVLVPISPRLSVSTTSKGVVVEKLPLHTIKPIEFKENLIKSPSCSQPP
LLQLNSSMNFSGIMEHQTA

>Strigamia_maritima_ACPR (from WGS, AFFK01021969.1 and AFFK01021968.1)
MNDCTAAPASNSCTHPIMDWNLTSSEMTFSEQIRPNLSLTLPLHLTFTRDSLIRVSVYGILFVVAA
VGNLTVLITLLKNRRRRSRINMMILHLAVADLIVTFIMIPLEMGWRLTVQWVAGEVACRTFLFLRA
FGLYLSSMVLVCVSLDRYFAILHPLKVNDAQRRGKIMLLFAWTISILCSVPQSVVFHVQRHPDYPF
FEQCVTFNSFPTDNHELAYNMFCMLAMYGVPLLVIVFVYTRILCEISNKSRETRDELERDGRIRGR
FRLRRSDMTNIERARARTLRMTIIIVIAFIWCWTPYFVMTMWYMFDRESATQVNQNSVDERLQESL
FMFAVSNSCVNPLVYGSYAVNFRREYTRCLCCKKNLRRKTPVPA

>Tribolium_castaneum_ACPR (ABX52400.1)
MQAVGKMGEEHYDEDSKSNFSVLNETLDGFANETVSPDVLFQQNLTVILVYSALFVVAAVGNLTVF
ISLFRSRHRKSRISLMIRHLAIADLIVTFIMIPIEVGWRLTGKWIAGNVACKVFLFLRAFGPYLSS
NVLVCVSLDRYFAVLHPLRVNDARRRGKIMLAFAWGTSFVYCIPQSFVFRVRAHPKYPNYEQCVSF
GFFENTAQEIAYNLMCVMCMYFIPLFVIIVAYTAIMCEISKNSKETKGESYRTSNGRMRLRRSDIS
NIERARSRTLRMTITIVAVYVWCCTPYVIITMWYMFDRASATSLPXWLQDTFFMMVVSNSCMNPIV
XGSYVINFQRVNCNCFCFXKTASESHLNVGSGATRSTAMVHGAGGNGYTRSPTPKSNLNLTGLLSK
SRLPDKPPSVGHISFLSEPRTARNYRSSFHSEPCSRTRMCPDELCLDTSCHSADYYSSAVL

>Aedes_aegypti_CRZR (from WGS, AAGE02020874.1 and AAGE02020871.1)
MATNLSSILFESLYTNAATATAANGSPSDSYTTTDAFIGTQWTPAAALGSLLATERSETSPDGANG
TGWNKVPHQQPLLLVPSYLSTLATMAANIVEANTTTNVTLSYEDCSLLNVTYGTDGLSTSHPWILC
LPHAPTLSKTGVIRVVVLSAMAIVSLLGNIATMWSIRKNRKSRRLARHNWSAIYSLIFHLSIADLL
VTVFCIIGEAAWSYTVEWVAGTVACKLFKLLQMFSLYLSTYVLVLIGVDRWVAVKYPMKSLNTARR
CHRFLCGAYSLSFLLSIPQWMIFRVAKGPFLEDFYQCVTHGFYTDRWQEQLYTTFTLVFMFIIPLL
ILIGTYLSTFRTISSSEKIFRIETSAVDRSYYRRSDTNRQRLIHKAKMKSLRISVVIVVVFIVCWT
PYYIMMLIFMFLNPTERLGEDLQTGIFFFGMSNSLINPLIYGAFHLIPIRRRRNQYNQHVREGSVY
FQRSSTCNNQNGNHHQQLLQQPASPPQLELHSQHHHHHHSHHHQPVQPRNSYLAANHKMSHSHSNL
AEEISLMALEKDLKSLDENVNQINLKGTSGGKRSLARKFYSLSQLLRRTRPTKL

>Anopheles_gambiae_CRZR (AAQ67361.1)
MPPSFNLSTVTYELISGNVSAALENFTASMAGAFPFEDLHDPASSIARNASFTLGLGLANVIQLQQ
QQQQQQLHHSPHQYHQQVQHQPQPPSTPFANVSTGQNESLANLLLHPGVHQLSSGLVTLVGDIMAQ
SGDTILPREECDRLNISYAFENGTALEIPGLSCYEHAPTLSKSGVIRVIVLSAMAIVSLLGNVATM
WNIQKNRKSRRVTRHNWSAIYSLIFHLSIADVLVTGFCLIGEAAWYYTVDWVAGNLFCKLFKLCQM
FSLYLSTYVLVLVGVDRWVAVKYPMKSLNTARRCHRFLFVAYLLSFLLSTPQWMIFRVAKGPFVED
FYQCVTHGFYTDRWQEQLYTTFTLVFMFIIPLLILIGTYLSTFMTISSSEKIFRIDTSAVDRTTYY
RRSDTNRQRLIHKAKMKSLRISVVIVVAFVVCWTPYYIMMLIFMFLNPTERFGEDLQSGIFFFGMS
NSLINPLIYGAFHLVPIRQRRNQYNQHVREGSVYFQRSSTFNHHNGHQRNLNPHIKFSNSHSNLPE

EISLMSLEKDLRSLDENVNQIHQKGGGGGGGGGGGGGGVGGGIGLSLGGAAGVDGSRRIKRRSFAS
KFLSFSRLLQRNHPTKL

>Apis_mellifera_CRZR (NP_001137393.1)
MTILNNYTTYMLLACDNLTNFFNHTRDLTLDSRLWNTSELSHPFHSYNLKNITCLEQAPHSNGNTL
FKSLILTIMAVVSILANLATIYSIVRCRRRHHSWSAIYTLILQLAVADLFVSVFCIGGEAMWNYTV
EWIWGNVACKLFKFFQVFSLYLSTFVLVLIGVDRFFAIRYPMKGMNTADRCLKFIVAAWILSFVLS
LPQIIIFRVVQGPFVEKFEQCVTYGFYTEPWQEQLYVSFGLFSMFLLPLGILIATYVFTIITISRS
ERMFKVKLANNDICHVNGDVNRRKLMYRAKAKSLRISIVIVTAFIFWWTPYYTMMIIFMFSCPDKH
VSDELQNIIFFFGMSNSLVNPLIYGAFHLWPRKRRNFMHREISTTQRRLTPTSYGSSYRRDSRETR
IPILPKN

>Aplysia_californica_CRZR/GnRHR (XP_012940604.1)
MDGTDSDGSILQRANQAFLASSSGPLQLPIPYNFSAQNSSVHRKRHDVYSPRSAHHDFILASDNIS
SLLLNTTAPNMISNNSSFEFQFLNTTHPSNVTFENAPTFTTTSLIKTIVFGIMFGISFVGNMATIV
QMRRLRRRKSTINTLIVNLALADLLVTFFCIAGEAAWTVTVQWLAGNVMCKFVKYMQVFALYLSTY
ITVAISLDRCVAILDPMRRNGAAQRVRTMIVFAWIFSALFSIPQPIVFNVLRGPFKEVFYQCVTFG
SYDSAWQLQMYAIASLMLMFVLPLAVMGTAYGLIFTTISRKSKEHSVMDSIQMPARKTWVTRWVNY
KRYLCMAFCSKPISRRSSIMSNYSEDHAARGPVRSYLLRKAKRKSLIMSFVIVLAFMVCWTPYYII
FICITFLDKVIDPVIFNYFSFIGLSNSMLNPMIYGAFQLCKVQFYNPSSWRRNVWRGTSPKLQSSN
PNRRSDFPPGTPASSSISRDSRNRLLGQGQGCHCQTCHYEAKEQKVCIPALDRKAQASKTSTKSSE
RCGKFTHKHFTNGEVKHCLCFHSY

>Bombyx_mori_CRZR (NP_001127719.1)
MDNEGNSTILYDANIMYPSELTLRTEFNTDGNNMNVCAIWPIEKCIEILKLNDTKTDDILGRSFIY
NDTQLTCLEHAPVLTKTTVIRASVLSAMAFLSFIGNVATIISIRRSKRCRGRARPSWTAIYSLIFQ
LSIADLLVTIFCIAGEAAWSFAVQWYAGNIGCKLFKFLQMLALYLSTFVLVLIGVDRWLAVKYPMK
SMATATRSGRLVIIAWVLSVILSIPQAVVFRVAKGPFFEEFHQCVTHGFYTERWQEQAYTTLSLVF
MFILPLIILVSTYVSTVRTIAQSEKVFKPEVRRQEKYFTPDMNRRRLIDRAKMKSLRMSVVIVAAF
LIWWTPYYVMMIIFTFLNPDKNQSEELLNGIFFFGMSNSLVNPIIYGAFHLWPRKKRSYQHSDRES
GGHHASILRRGDNNTSSVRLTTIRSLRSSAKYSNGQNISLL

>Culex_pipiens-CRZR (from WGS, AAWU01031675.1, AAWU0103176.1,
AAWU0103178.1 and AAWU01031683.1)
MAANLSSFLYESFYGGNATVLDNYTSSTPYDTFQLDSGWPLTTPTVSSTFFPSAEEDNGTGLHQLS
TYLSANLTGIVAVYSQEVCQLQNITFTPDGASSSHPGVFCLPHAPKLSRSGVIRVLVLAAMAIVSL
LGNIATMWSIQRNRKSRRLARHNWSAIYSLIFHLSIADLLVTVFCIIGEATWSYTVAWLAGTVACK
LVKLLQMFSLYLSTYVLVLIGVDRWVAVKYPMKSLNTARRCHRFLLCAYSLSFLLSIPQWMIFRVA
KGPFLEDFYQCVTHGFYSERWQEQLYTTFTLVFMFIIPLLILIGTYLSTFRTISSSEKIFRIETSA
VDRSYYRRSDTNRQRLIHKAKMKSLRISVVIVVVFIVCWTPYYIMMLIFMFLDPTDGLVEDLQTGI
FFFGMSNSLINPLIYGAFHLIPIRRRHNQYKQHVREGSVYFQRSSTCNNQQPNGTSPHQLQPSPTP
VPPLRSPGSVPLGPLVGTPPIGITSKQLAVARSNSNLAEEISLMSLEKDLKSLDENVNQIHQKNSA
AASSRRSLARKFITLTQLLSRTKPTKL

>Daphnia_pulex_CRZR (EFX87464.1)
MTVYHINQSDPNILIPYVITSKDFRDNSSLLEEEAPTLTTSASVKATVLCVMAVASLIGNLATLIS
IAVSKKGTSSSLYTLLFQLAISDLLVSVWCLSGEAAWTYTVEWKGGQFLCKTFKFSQVLIGFDRLR
AIRRPISHRSCLKPICVAWILSAVLASPQLLIFSVLRGPFRELFYQCVTYGFYTEEWQEQLYTVFS
LLTMFVIPLIVLIGCYVCIFCTIAKKERAFVIRAPHPSMERWPAESAAVSHFRTRDCQQHRQHSPQ
RHQHTSFNRRRNNIFKRAKMKSLRISIIIVTAFVICWAPYYFMMITFIFLNPDDKLGEDMQSAIFF
FGMSNSLVNPLIYGAFHLWQPNIGRSRKLPGSFSTAFTDSVRAGLIVGQRRRPAEPAADNAVEVAF
LGGVLISTSEAAGVVGRDRRSFFDGPKKPKFPSIVLVGLVDQPVPTIVGMPQQNPYPGPPPPSYAP
PSYPAPPSYSPPSYPPPSYSPPAYAPPPSPYAPQSYMAPPPPYQPPQYAPAAPAPMHPPQAQQLPA
PELQQHEQSVPVPAQEDLKPTEADPPAPAAKNVETESSDAAPSTESTNSDGKSE

>Drosophila_melanogaster_CRZR (AAM21341.1)
MEDEWGSFDRLPSVPSASMDLETENEVVSNWSTLANFTRLVAGAAPEIVNYTLNMIDVGVGMATDI
SNLSVSTTPLPAYAISNSSSLAHTNSRHEAPPMAEQVPEHVMDHAPQLSRSGLLKVYVLAVMALFS
LLGNLLTIWNIYKTRISRRNSRHTWSAIYSLMFHLSIADVLVTWFCIIGEAAWCYTVQWLANELTC
KLVKLFQMFSLYLSTYVLVLIGVDRWIAVKYPMKSLNMAKRCHRLLGGTYILSLVLSLPQFFIFHV
ARGPFVEEFYQCVTHGFYTADWQEQMYATFTLVFTFLLPLCILFGTYMSTFRTISSSEKMFQGSKL
ANYSTAKLPTQTNRQRLIHKAKMKSLRISVVIIIAFLICWTPYYVMMIMFMFLNPDKRLGDDLQDA
IFFFGMSNSLVNPLIYGAFHLCPGKGGKSSGGGGNNNAYSLNRGDSQRTPSMLTAVTQVDGTGGSS
RQMRAFRQQSYYRSSSNGTAGPGAAPFKEQVGLLHVGPGNGTPGGSVSSGATPQLIRKGSALLARQ
PSCLREQEHQQRLLLHEKPSTLVLSYDSQRGGVGVGVASGLLDNNERVSSV

>Ixodes_scapularis_CRZR (from WGS, ABJB010847221.1 and
ABJB01088645.1)

MWLSCRLGCVVLLAATVLSSVAIALDSGGNSTLPGDECAPGNATCGTEPLHAPVFQPSSLIRVVIL
VLIGVLSLVGNCATLVSIWKTRLRARSTVYLLLAHLSVADLLVTFFCVLAEAAWTWTVQWTAGDGA
CKAVKFLQMFSLYLSTFILVVIAFDRFAAIRFPMRRASARRTVVRMVFGVWALSAMLSLPQVFIFR
VQRGPFEEEFYQCVTYGFYSAQWQEQLYTTVSLVLMFLLPLVTLITTYICTFYTISIQRSFFVPSK
DGASGSGKHTQENHEPQARRHAKAETLAAPTHSAMDDARRKLLHKAKMKSLMITVVIVLAFIVCWT
PYYCMMIIFIFLDPDDQLTEELQAGIFFFGSSTAMINPLIYGVFHLRRRPSRGSKQFNSSVASRGA
ENSVLLTNCPRRTRSGHQPPQQLRMVQQTSHPVVRLKYSVVSNGCLKQKQATVVDVEDIDR

>Nasonia_vitripennis_CRZR (from WGS, AAZX01006363.1,
AAZX01010633.1, AAZX01000283.1, AAZX01013590.1 and AZX01015155.1)
MYVLEEEPCLNIRNASGLISTNILRNSSCLGHAPQLTYGAYLRAVVLLSMTLLSFLANLATIWSIK
SNKRKSQNCSAIYSLILHLSVADLFVTVFCMGGEALWSYNVAWIWGNTACKAFKFLQMFSLYLSTF
VLVLIGIDRFVAVKYPMKTLNTAKKCNQLISFIWFISFILSTPQVVIFHVAQGPFIEDFSQCVTHG
FYTEVWQEQLYTTLSLIFMFIMPLTILITTYMSTVITIARSERLFKSELANSSSAHKTGDVNRRRL
IHRAKTKSLRISVVIVVAFVLWWTPYYIMMIIFMFLNPDKHVSADMQKGIFFFGMSNSLVNPLIYG
AFHLWPQKKNRKHRENSIMQLRSTTTNASLMMDHRSTNARFTKQTKYSHIQTHEGLSVNHDTVVVH
LIENSEKNKFCGRAAKIILHYNLPTKSN

>Strigamia_maritima_CRZR1 (from WGS, AFFK01019957.1)
MLELDMNGTLNASQLLEAPAFSKQRLIKSLVLAAMAFVSLVGNVATLVNLSRSKRRTVSTMYMLIT
QLAIADLLVTFFCLVPESVWAATVQWLAGDALCKIIKYLQMFSLYLSTFVLVLIGIDRFIAIRLPM
DRSDAHRKCNLLIVCVWLISALLSIPQMFIFRVRRGPFVQDFYQCVTHGYYTTEWEEQVYTTATLV
LMFILPLLTIVTTYICTFCTIARNEQVFREYDSDKQMHVSRQKLFRKAKIKSLQITVVIVLAFVVC
WTPYYTMMITYIFLDPVSHLSQEVQAGIFFFGMSNSMVNPVIYGAFHLRKRGGKRRYLSSRFQSQS
TASVRKKKDTSTKGVSFAPDPETTCGVTVVEEVVEEVVEELDLNHFDNRSNSNDDFAKTTTNI

>Strigamia_maritima_CRZR2 (from WGS, AFFK01020014.1)
MEDINRTNTSVLMATAPAFNEQRLIKSVVLAVMAVLSLIGNVATLINLYRSKRRTVSTMYMLITQL
AVADLLVTFFCVVPESVWVATVQWLAGDAMCKIIKYLQMFSLYLSTFVLVLIGIDRFIAIRLPMER
SNAHRKCNVLIGCVWVLSALISIPQVFIFRVRRGPFEEDFYQCVTYGFYTTVWEEKLYTTSTLVLM
FILPLLTIITTYICTFYTIARNEQVFRECDSDKQMHVLRQKLFRKAKIKSLQITVVIVLAFVVCWT
PYYMMMLTYIFLDPEAQLSQEVQSGIFFFGMSNSMVNPVIYGAFHLRKRGGKRRYQSSRFQSQSTI
NLKKRKNNAASSALETTCGVSVVEEETVLNHFENRCQDEFVNAKTTSNI

>Capitella_teleta_CRZR/GnRHR (ELT93721.1)
MDSKWIPDITINNKSITAFELSFLSSIRPELFSKNFTSFNRTAVQLLFSNHTFVAYFLEKLPRIPI
FNEMSMIKTVVLSCMFVVAFIGNTATLVQMYRMRRRKSTINLLLLHLAIADLLVTFFCIVSEAVWA
STIQWFAGNFMCKLVKFLQVFGLYLSTYIIVIIALDRCLAILDPMSRNRAPRRVRTMITVAWIMAG
FFSTPQIIIFKVERGPFPEDFFQCVTYGTYSQPWQEQLYSCLSVGFMFIIPLVAMVSAYMLIFHTI
AKKSRDFQRAASDMLSRGPVRGQLFRKAKVKSLRMTAVIVVAFIVCWTPYFVILILHTFSSNKLDE
KSSLWIFFFGMANSMINPLIYRAFQISKNRKRKR

>Crassostrea_gigas_CRZR/GnRHR (from WGS, AFTI01018035.1)
MNYSMSSFAIGNPPEVPYNSHGSSSTALTNVLLNGSEPRPNMTGDNALSTTESPMPPTFQDSLVKT
VVLILLFTISFIGNTATLIQMYRVRKRRSTINTLIVNLAVADLIVTFFCMAAEAVWALTVQWVAGV
AMCKMLRFIQGTGLLVSTYITVVISLDRCCVIIDPISRNKAPQRVRIMIIMSWFLSALFSIPQALL
FSVHRGPFKEDFYQCVDILGYPSPAYKKLYNIFCMIIQFFLPLTLMIIAYGLIFFTISRKSKEFGE
PENVSNSSDQREHSGRSHVRSGLLRKAKRKALRMSLFIVIAFFVCWLPYYVVFTGFAFGHWTSLDP
SLMSGLTFVGLTNSILNPIIYGAFQLCKVPNNPRLVWRTIVSKPSSNDFMYILPMEIKMGNKGRLR
IRSNSNQLYLQTSSS

>Lottia_gigantea_CRZR/GnRHR (ESP05621)
MNYFEVIGNDSSINRSGNWGIIIPEPQPPTFDDIAMTKCIVFGTMFVIAFIGNVATLIQMYRMRHR
KSTINTLIVNLAIGDLFIVCFCFPVEGIWAATVQWYGGTAMCKIIKFIQVFSLYLSTYVTVAIGLD
RCVAILDPMRRKSAPTRVRTMIMLAWFFSALFSIPQAIIFNVRRGPFKEDFYQCVTMGSYPNAWQE
EVYSIASFLLMFVIPLVIIGTAYGLIFSTIARKSKEFIKKNPTVRTTSYNDIQRGPVRSNLLRKAK
RKSLRMSIVIVVVFVMCWTPYYVIFICLTFLDWEEINPRIYRLVLWLFFLGMSNAVMNPLIYGAFQ
ICKVHTPRSVHLRAFRSHKILLLFYSKFYDGFMFG

>Octopus_vulgaris_CRZR/GnRHR (Q2V2K5)
MDYLNDSMFNNMTYNITSTPLPDAPRFDNVYVSKLCVLGTVFVISFFGNTLVIIQIFRIRGSRSTI
QSLILNLAIADLMVSFFNILMDIIWSATVEWLAGNTMCKIMKYLTVFGLHLSTYITVSIALDRCFA

ILSPMSRSKAPLRVRIMITMAWVLSAIFSIPQAVIFQEQRKMFRQGMFHQCRDSYNALWQKQLYSA
SSLILLFVIPLIIMVTSYLLILKTIVKTSRQFHDTPISPTSMSCYSVNHGQIRTHLFERARKRSSR
MSAVIVAAFILCWTPYYIIFLGFAFFQWDNSRTVIYFFTLGTSNCMLNPLIYGAFTIYKVHRGRSG
SANSPSGTRLMIMVNKRGRSTTTTTNRMSGSGRRQLTTGQTITQCASLTNPHQPVRPSPGINSTTS
PNGKMPTKPPG

>Ciona_intestinalis_GnRHR1 (NP_001028997.1)
MMTSDISQGATNIDNNWTSTAAVLGLNSTVNSTALPCEAHDVLIQTWFQFDTLHLVRVLVTWLLFC
LSMAGNMFVLWSLRGSKSRHFIMFHLALSNLIYTIFVMPSDAVWNTTMEWLAGDVMCRLCQMMKQF
GMYASSFMVVVIGADRVTGILSPLPCHSQRKRGYYMVATAWISSLICCLPAGFIFSVASIPTCEGI
PIYQCIDFNVLQDVSLLRPYYFFTMCMSFLLPLICTLVSYSLIVCEISTMKERDRVLMGRRHSVNT
ASIQRAKNRTILMRTLITLTFLVCWGPYYGKGIYDWFIRYEDHTPPDAWDTVMYVVMYLNPVLHPI
VFGVFLKEIRGKFKQRLNCARKRFFKQGDFKTVPNAQSSMNYSIASVLNRPRRMSSTSRGSFSSYA
TGATHLNGSSHVTINGQCSNNGSNGSIKTQPQFFGANRMVAPQQQLLLSSESAL

>Ciona_intestinalis_GnRHR2 (NP_001028996.1)
MTTAAFTQDYVDGIYPSTATDTFMPMCQSHDTIIQTWFQFDTLYLVRVLVTWLLFCLSMAGNMFVL
WSLRGSRSRHFIMFHLALSDLIYTIFVMPSDAVWNTTMEWLAGDVMCRLCQMMKQFGMYASSFMVV
VIGMDRVTAILSPLTHEGQRKRGYCMVLAAWTTSLLCCIPAGALFSLLTVETCEGINVYQCVDFNI
VKDRSLLRPYYFFTMCMSFLLSLICTLVSYSLIVCEISNMKERDRVLMGRRKSVNTASIQRAKNRT
ILMRTLITLTFLVCWGPYYGKGIYDWFQKPAIGPPAPLDTAMYIVMYLNPVLHPIVFGVFMKEIRS
KFKKTSCVAGLKRKRVQVRSGSVSRLSLTSYATGLTQVQYLSNGAAETTVLTGATQQVPSLDESVH
TKAPE

>Ciona_intestinalis_GnRHR3 (AAW70562)
MATVSSLVTTTATVMDGTTMSTHITSTFDINASYHSLNSTLDPNSTYFEKWCPYYHKMLTFNTIQL
TRVIITWILFLVSTCGNSFVLYCLCMRKQRLHVHVITMHLTLADLAFTFFSMPMDATWNTTMAWLG
SEFLCRLCQFLKQFGMYISSLMVVVIALDRVFSILSPMSANQQRKRTKILLISAWTLSLLCAIPAL
FLFSLIKKQFCPDQPIFYQCVDFSPNINKQDLKPYYFFTMCVSFLIPLFFTVISYSLILCEINAMQ
RRDERITGRRDNNIERARMKTLVLTSLVTLSFIVLWGPYYAMGIYHWFNPRERATFPKEISVGLFV
LMYFHPAVHPILYGFFMKDIRKHFLVTLMRCFKLSRIPASRRASEKFGSCQRHLPEGNPHPRAGRA
ASSPLLSPVTERTVLRATSVPFINAGKCNKNNNRQEIFLQVPPTNGELSTKRCCDERV

>Ciona_intestinalis_GnRHR4 (NP_001028994)
MMTSPTMTSNITGCNVTSTYSPEFIIENQYDCDAMFESFLSSQRLVFDSYHLTRIWVTWVIFFISL
AGNLTVLISVTVLRKTQSYSHCQLIMTHLSLANLAFTLFVLPMDAIWNYTLEWLAGDVMCRIMNSL
KQFAMYISSAMIMVMGVDRVTGLLRPVSANQQRRRIVKFLTVAWVFSFINSIPPSVMFSAGPYWPM
RCECPNHYVVQCVDFHLIKKGREIFYIYSMFISFFIPLYCIIICYLIIAFSIAKMAKRAKATELQS
SGFRPPSSRKSLARRSLQRAKKISQLVTGLITITFVICWGPYYVVGLMHWFNEQHIERLPEGIMLM
SLYLNPCLHPFITVCLMKEIRESICRKPNCSLATPR

>Danio_rerio_GnRHR1 (NP_001138452)
MSGNVSLSLISLLENSSLASSSSPQSPQWETPTFTTAARYRVVATLVLFVFAAVSNLSVLISVTRG
RGRHLASHLRLLIASLASADLVMTFVVMPLDAIWNITVQWYAGDAMCKLLCFLKLFAMHSAAFILV

VVSLDRHHAILHPLEALDAGRRNRRVLLAAWILSVLLASPQLFIYRALKAEGVDFVQCATHGSFRH
RWQETAYNMFHFVTLYVFPLLVMSFCYTRILVEINRQMPRGKGKGGEPCLRRSGADMIPKARMKTL
KMTIIIVASFVVCWTPYYLLGIWYWFQPRMLQVTPVYAHHALFVFGNLNTCCDPVIYGFFTPSFRA
DITSCFSRRNQNCSPKSLDRLSARRGGASGEAESDPGSGDQPSGQTA

>Danio_rerio_GnRHR2 (NP_001138451)
MNTTQLIEDLLQNSSCKHEAKHLNSNIAGDLPHPLHLPRFTAASQVRVTLTLVLCALSACCNLAVL
YSANNNQRKRSHVRLIITNLAAADLLVTFVVMPTDAVWNITLQWRAGDLACRTLMFLKLVAMYSCA
FVTVVISLDRQAAILNPLSINKAMRRNKVLLGVAWTMSVVLSIPQVFVFHVVEIDSPKQFVQCTTY
GSFSSRWQETFYNMFTFTFLFLLPLFIMISCYTRILFEISKKMTEDRLLSNKVQLRRSKNNIPKAR
MRTLKMTVVIVLSFMVCWTPYYMLGLWYWFSPAGLEETVSQSLSHILFIFGLLNACLDPIIYGLFT
VPLCRGMRHRQREQNIVAFELENTNNSLMTSIRTSTSPLTLKRLAVQQTSREKLEWDAEFVLKTGD
DGTKNALYSSGVATVE

>Danio_rerio_GnRHR3 (NP_001170921)
MSGNWSQYNASLLPVWTAPSFTPAAQARVAATALLFVFAAGSNLALLVSVCRSRRLASHLRPLILS
LAAADLLMTFVVMPLDMVWNVTVQWYAGDVVCKLLCFLKLFAMQTSAFILVGISLDRHQAILRPLD
TLTAPQRNRRRMLTAWSLSALIASPQLFIFHTVKAKSVDFTQCVTHGSFSERWHETAYNMFHFVTL
YVIPLLVMSCCYTCILIEINRQLHNSNKGDSLRRSGTDMIPKARMKTLKMTLIIVLSFVVCWTPYY
LLGIWYWFQPEMLTVTPEYVHHLLFVFGNLNSCCDPVIYGLYTPSFRSDLIRFCCCRHHKNTPRPQ
QPTRHT

>Danio_rerio_GnRHR4 (NP_001091663.1)
MNDSSPTSENIMFHQLTADTLNGSCDLPTCNNNTGEAALQLPTFSMAAKVRVIITFTLCAVSAVCN
LGVLWAASTNNKRKSHVRILIMNLTVADLLVTFIVMPVDAAWNITVQWLAGDLACRLLMFLKLVAM
YSCAFVTVVISLDRQSAILNPLAINKAKKKNKIMLSVAWAMSVVLSVPQMFLFHNVTITVPANFTQ
CTTRGSFVKHWQETIYNMFTFICLFLIPLAIMICCYTRILVEISRRMTKGNISSKEVHLRRSHSNI
PKARMRTLKMSIVIVTSFIVCWTPYYLLGLWYWFLPEDLEETVSHSLTHMLFIFGLFNAILDPITY
GLFTIHFRKGLKRYCRSAVVLTESENNSIITGSLKCSPSPFRMKRVTQSGTGTDPKQNTSTVGEED
KKAADGKTKE

>Gallus_gallus_GnRHR1 (NP_989984)
MCVPAALIEAEPPHHPTTEGDTNTSATHCLEHWVEPRFTKAAKVRVAITAVFFLLAACSNTAVLGS
LLRKRRKCHVRPLILSLALADLLVTVAVMPLDAAWNVTVQWYGGDLSCKLLNFLKLFAMYAAALVL
VVISLDRHAAVLQPFARARRRNGLLLRAAWLGSVLLASPQLFLFHVHTVPGGNFTQCVTHGSFRAH
WEETVYNMFTFTTLYITPLSIMIVCYVRIIWEISKQLKINKSLVRSQNDHISKARMKTLKMTIVIV
ASFIICWTPYYLLGLWYWFHPAMIQRMPEYINHSFFLFGLLHTCTDPIIYGLYTPSFREDVQLCLR
GIEAAISQHVRHKPISVSEKTTKDGDVNGQVTSGGSNGTTVNTVC

>Gallus_gallus_GnRHR2 (NP_001012627.1)
MARLGGGTGQDAAAAGGGWLDPGPTVGNVSTEPSSSTSHPKRGCAWSPRLESAEEPLLLPTFSPAA
QARVAATFVLFVLSAGCNVAVLRAAGGRRGGGRSHIRVLLRHLAAADLLVTVVVMPLDAIWNITLQ
WRAGDLACRLLMYLRLLAMYASAFVTVVISLDRQAAILRPLAIARARCRNRAMLRAAWMLSAALAV
PQLFLFHTVTLHAPHNFTQCTTHGSFPQPWHETLYNMLSFSCLFLLPLLIMVCCYTRILLEISRRM

GSSLFSSRDVPLRCSGSNIPRARLRTLKMSLVIVSSFILCWTPYYLLGLWYWFCPRAMQQKVPPSL
SHILFIFGLFNACLDPITYGLFTIPFRRRCGCPCGHSSEPEPPSPATGSFHCSASSLRGRQGMGGT
EGPHPPIELGLPTGAGSCQSSAL

>Homo_sapiens_GnRHR1 (NP_000397.1)
MANSASPEQNQNHCSAINNSIPLMQGNLPTLTLSGKIRVTVTFFLFLLSATFNASFLLKLQKWTQK
KEKGKKLSRMKLLLKHLTLANLLETLIVMPLDGMWNITVQWYAGELLCKVLSYLKLFSMYAPAFMM
VVISLDRSLAITRPLALKSNSKVGQSMVGLAWILSSVFAGPQLYIFRMIHLADSSGQTKVFSQCVT
HCSFSQWWHQAFYNFFTFSCLFIIPLFIMLICNAKIIFTLTRVLHQDPHELQLNQSKNNIPRARLK
TLKMTVAFATSFTVCWTPYYVLGIWYWFDPEMLNRLSDPVNHFFFLFAFLNPCFDPLIYGYFSL

>Strongylocentrotus_purpuratus_GnRHR1 (NP_001116990.1)
MAVPTSHRDGSEPGMNAEWGKPGGFDGADVVIIAPTMDIGFAYNYSLNDSLDASGNETDFEVPVFT
PEYLIRVVTLTSIMVLSGPLNVAVFASLWRERRRKSRINLLIMHLTLADLLITFINIPTDVIWFCT
VRWLAGNVMCKLLMFIESSAMYASSFVLIVISLDRFAAIVHPLSVSKADRRCKIMLRVAWSSSVFC
SIPQLFVHEVGSPPEDPSFTQCVDYNFANNRPRLWWLYHVFVTLAMYIVPLIAIFGCYVAIVYKIC
KKSREVTKALKNGNHPGLRRTGLDRLPRARMKALQLTATIVTAFIICWSPYYIVSTWYHFDSGWQS
EEKKMLKPEWVIDVLMALGYSNICVDPIIYGLFSTKLWRQLRSCWKKKSLSDSRSPKTMTKRTLSS
SHSTVTRATSVRFGVMTSVHSCPHEHPGIEMSHMTQTRVDSVSD

>Strongylocentrotus_purpuratus_GnRHR2 (NP_001116992)
MTRTTYLSPVTTESAIIHRLNISLFEGLSNYTTAANFSDYDFSYFTESPDSNSSISKTPTFELAYL
IRIITLSTILVLSVPLNFIVLLTVWRQRGRKSRVNLLVLHLTIADLLITFINISTDVVWFCTVAWL
AGNTMCKLIMFMETFAMYASSFVLIVISIDRYAAIVHPLSVRAADRRCKIMLRVAWGSAAVCSIPQ
LVVHELMSPSFNPTFYQCVDYGFKEKYFRLWWLYHIFVTLIMYVVPLATIFACYSSIVWKIVRRTN
GLTSWSKKPGKTTLRMAGNDGIPRARMRAFRLTAAIVGGFVICWSPYYVVSTWYHVDHGWQDESEP
PDPPWLFDVLIALGYLNTCVDPVLYGIFTTKLARDVRHCWRTSKPVARGRQRLTSRRSTSSSNNNP
VTVGSSVRLGMTTVNVVSEERQAVHFSIPDEDERTVIP

>Strongylocentrotus_purpuratus_GnRHR3 (NP_001116991)
MLDLARTLKSPKSATIMSSFYIPDVGSDYANVTTNYTYMDSGSAEIFAESLFRILTLSAIFLISVP
LNIIVLIIVFRRRRHKSRVNILVMHLTLADLFVALINIPTDVVWFYTVRWLAGNAMCKIIMFIESF
NMYGSSFILIVISLDRYAAIVHPLSIHQADRRCKIMVRAAWTCSALCSVPQFLVHEVLSPYEAPDF
TQCVDYRFAFEHPTLWRLYHVFVTLAMYVVPLITIFTCYAAIIYKICRKTKQARQSINNNESSVLR
RSCSSSLPRARMKALRLTATIVTAFIMCWGPYYVLSTWYHFDIRWQTGAGLPNPDWMIHIMMALGY
SNICLDPIIYGIFITRFSLCQRRGKSAVMHPLTTYRRSTMNSKVITNDGHTSSVRFNHTLVSSKNS
CSGTHAAVDTVTPLPDSDTRRNNFVNRRKTSTT

>Homo_sapiens_rhodopsin (NP_000530.1)
MNGTEGPNFYVPFSNATGVVRSPFEYPQYYLAEPWQFSMLAAYMFLLIVLGFPINFLTLYVTVQHK
KLRTPLNYILLNLAVADLFMVLGGFTSTLYTSLHGYFVFGPTGCNLEGFFATLGGEIALWSLVVLA
IERYVVVCKPMSNFRFGENHAIMGVAFTWVMALACAAPPLAGWSRYIPEGLQCSCGIDYYTLKPEV
NNESFVIYMFVVHFTIPMIIIFFCYGQLVFTVKEAAAQQQESATTQKAEKEVTRMVIIMVIAFLIC

WVPYASVAFYIFTHQGSNFGPIFMTIPAFFAKSAAIYNPVIYIMMNKQFRNCMLTTICCGKNPLGD
DEASATVSKTETSQVAPA

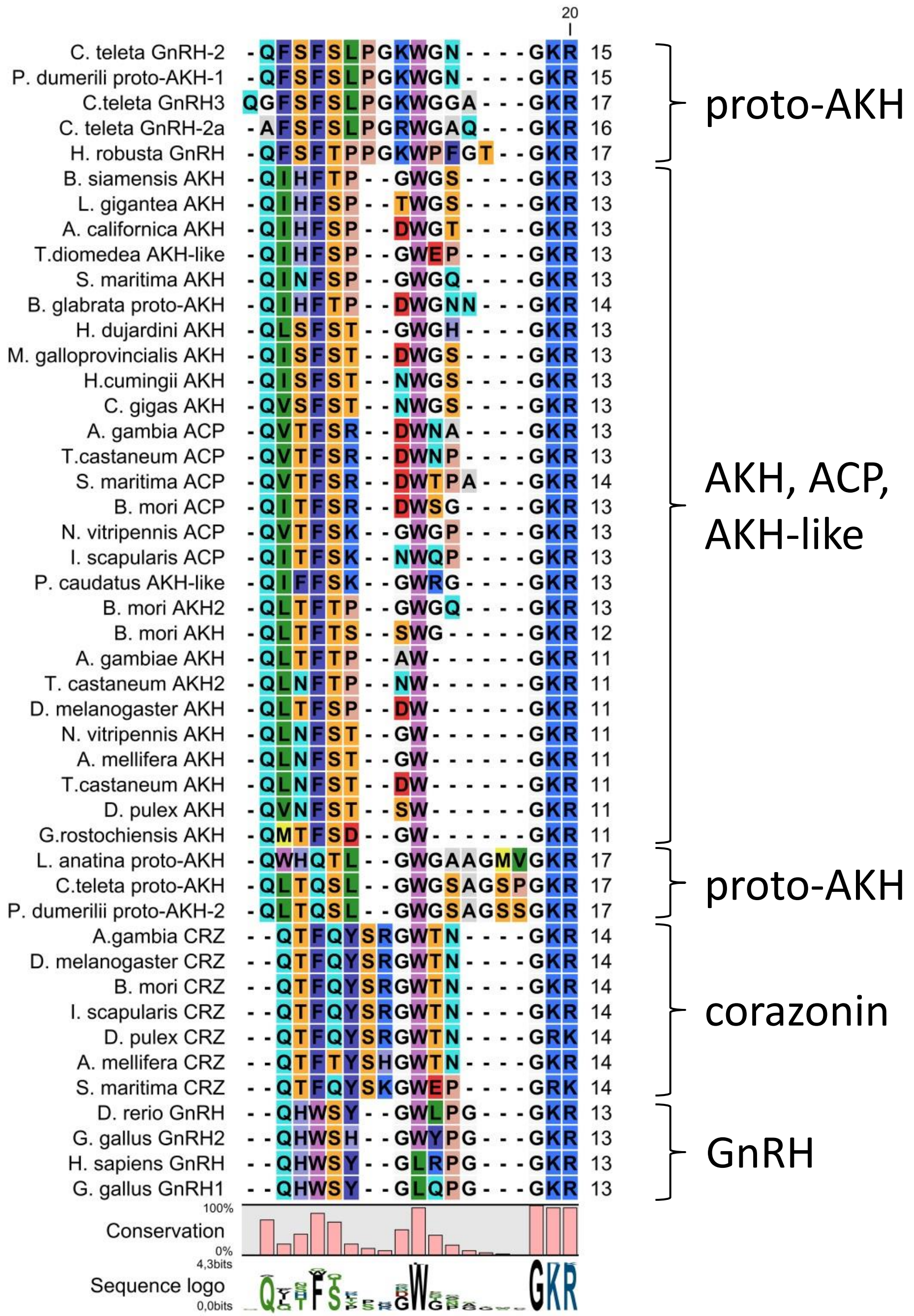
C. teleta GnRH-2
P. dumerili proto-AKH-1
C.teleta GnRH3
C. teleta GnRH-2a
H. robusta GnRH
B. siamensis AKH
L. gigantea AKH
A. californica AKH
T.diomedea AKH-like
S. maritima AKH
B. glabrata proto-AKH
H. dujardini AKH
M. galloprovincialis AKH
H.cumingii AKH
C. gigas AKH
A. gambia ACP
T.castaneum ACP
S. maritima ACP
B. mori ACP
N. vitripennis ACP
I. scapularis ACP
P. caudatus AKH-like
B. mori AKH2
B. mori AKH
A. gambiae AKH
T. castaneum AKH2
D. melanogaster AKH
N. vitripennis AKH
A. mellifera AKH
T.castaneum AKH
D. pulex AKH
G.rostochiensis AKH
L. anatina proto-AKH
C.teleta proto-AKH
P. dumerilii proto-AKH-2
A.gambia CRZ
D. melanogaster CRZ
B. mori CRZ
I. scapularis CRZ
D. pulex CRZ
A. mellifera CRZ
S. maritima CRZ
D. rerio GnRH
G. gallus GnRH2
H. sapiens GnRH
G. gallus GnRH1
proto-AKH
AKH, ACP, AKH-like
proto-AKH
corazonin
GnRH
Conservation
100%
0%
Sequence logo
4,3bits
0,0bits

Fig. S6

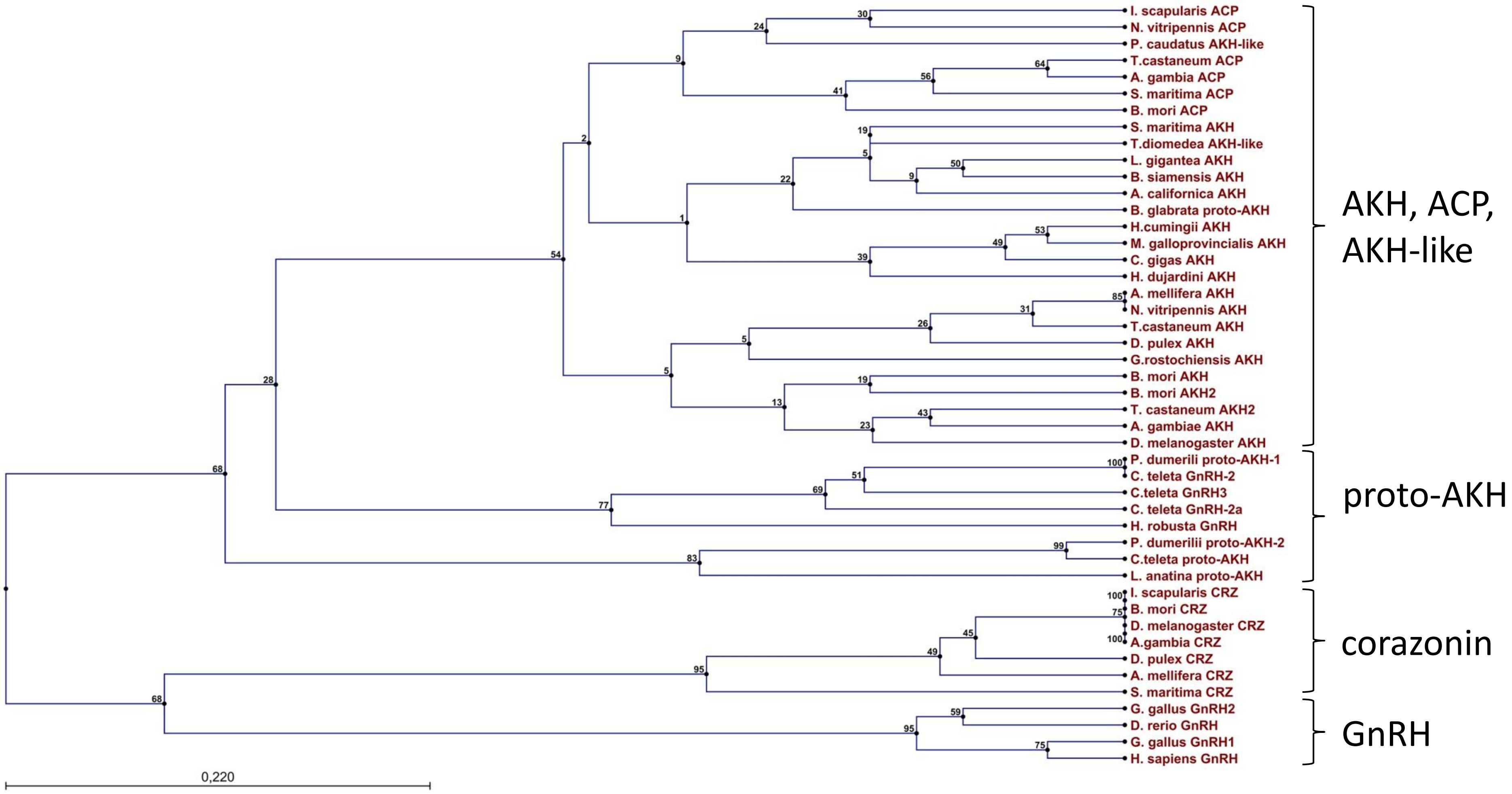
I. scapularis ACP
N. vitripennis ACP
P. caudatus AKH-like
T.castaneum ACP
A. gambia ACP
S. maritima ACP
B. mori ACP
S. maritima AKH
T.diomedea AKH-like
L. gigantea AKH
B. siamensis AKH
A. californica AKH
B. glabrata proto-AKH
H.cumingii AKH
M. galloprovincialis AKH
C. gigas AKH
H. dujardini AKH
A. mellifera AKH
N. vitripennis AKH
T.castaneum AKH
D. pulex AKH
G.rostochiensis AKH
B. mori AKH
B. mori AKH2
T. castaneum AKH2
A. gambiae AKH
D. melanogaster AKH
P. dumerili proto-AKH-1
C. teleta GnRH-2
C.teleta GnRH3
C. teleta GnRH-2a
H. robusta GnRH
P. dumerilii proto-AKH-2
C.teleta proto-AKH
L. anatina proto-AKH
I. scapularis CRZ
B. mori CRZ
D. melanogaster CRZ
A.gambia CRZ
D. pulex CRZ
A. mellifera CRZ
S. maritima CRZ
G. gallus GnRH2
D. rerio GnRH
G. gallus GnRH1
H. sapiens GnRH
AKH, ACP, AKH-like
proto-AKH
corazonin
GnRH
0,220

Fig. S7
